# Paired CSF–Plasma Analyte Signal Contrasts Across Alzheimer’s Disease Clinical Stages and MCI-to-Dementia Conversion

**DOI:** 10.3390/biomedicines14102407

**Published:** 2026-10-21

**Authors:** Yuepeng Deng, Dandan Chen, Haolin Yin, Zihao Lu, Xiaohe Tian, Qiyong Gong

**Affiliations:** 1 Department of Radiology, Huaxi MR Research Center (HMRRC), Institute of Radiology and Medical Imaging, Psychoradiology Key Laboratory of Sichuan Province, West China Hospital of Sichuan University, Chengdu 610041, China; 2 Xiamen Key Lab of Psychoradiology and Neuromodulation, Department of Radiology, West China Xiamen Hospital of Sichuan University, Xiamen 361000, China; 3 Department of Radiology and National Clinical Research Center for Geriatrics, Huaxi MR Research Center (HMRRC), Frontiers Science Center for Disease-Related Molecular Network, West China Hospital of Sichuan University, Chengdu 610041, China

**Keywords:** Alzheimer’s disease, mild cognitive impairment, cerebrospinal fluid, plasma, biomarkers, proteomics

## Abstract

**Background:** Cerebrospinal fluid (CSF) and plasma biomarkers provide complementary information about Alzheimer’s disease (AD), but matched analytes are commonly evaluated separately. We investigated whether relative CSF–plasma analyte signals were associated with AD clinical stage and subsequent conversion from mild cognitive impairment (MCI) to AD dementia. **Methods:** We analyzed paired CSF and plasma NULISAseq data from 1199 Alzheimer’s Disease Neuroimaging Initiative participants. For 125 shared analyte readouts, ΔNPQ was defined as the within-participant difference between CSF and plasma normalized protein quantifications. Clinical-stage associations were evaluated using covariate-adjusted models. In 547 participants with baseline MCI, we examined subsequent dementia conversion and compared matched CSF-only, plasma-only, ΔNPQ, and joint CSF–plasma representations. **Results:** Fourteen readouts mapping to eight gene-level targets were associated with more advanced clinical stage after false-discovery-rate correction, involving amyloid/tau, synaptic, astroglial, cholinergic, and neuroaxonal processes. During a median follow-up of 3.0 years, 203 participants with MCI converted to AD dementia. A cross-sectionally derived six-analyte ΔNPQ score was associated with subsequent conversion after adjustment for demographic, genetic, and baseline clinical characteristics (hazard ratio per standard deviation, 1.64; 95% confidence interval, 1.38–1.96). The joint CSF–plasma model yielded the largest estimated improvement in discrimination. After additional adjustment for amyloid/tau biomarkers, the association between ΔNPQ and dementia conversion was attenuated. **Conclusions:** Relative CSF–plasma analyte patterns are associated with AD clinical stage and subsequent dementia conversion in MCI. Paired-biofluid analysis provides a complementary framework for characterizing disease-associated molecular heterogeneity, rather than a demonstrably superior prognostic model.

## 1. Introduction

Cerebrospinal fluid (CSF) and plasma biomarkers have substantially advanced the biological characterization of Alzheimer’s disease (AD), providing complementary information about amyloid and tau pathology, neurodegeneration, and other disease-associated processes [1,2,3,4]. CSF biomarkers remain closely linked to established AD pathophysiology, whereas increasingly sensitive plasma assays offer more accessible approaches to biomarker assessment and clinical research [5,6]. Nevertheless, corresponding analytes are commonly evaluated as separate biomarker domains, even when measurements from both fluids are available within the same individual [7,8,9,10]. This may overlook differences not only in the magnitude of disease-associated molecular signals but also in their relationships across CSF and plasma. Characterizing these analyte-specific cross-biofluid relationships may therefore provide an additional perspective on molecular heterogeneity across the AD clinical spectrum.

Matched analyte signals in CSF and plasma are influenced by partially overlapping but distinct biological and technical processes. Differences in protein expression, release, metabolism, and clearance; preanalytical conditions; and assay-related matrix effects may contribute to heterogeneous relationships between the two fluids [11,12,13,14]. Consequently, disease-associated molecular changes need not be reflected similarly in CSF and plasma, and the direction or magnitude of their relative signals may vary across analytes. Paired-biofluid analysis may characterize these differences within individuals, complementing conventional assessments of each fluid separately.

Recent developments in high-sensitivity plasma phosphorylated tau assays and multiplex proteomic technologies have expanded the molecular processes that can be investigated across biofluids [15,16,17,18,19,20,21]. Recent paired-proteomic research demonstrated that the CSF–plasma protein balance varies with aging and cognitive impairment in a strongly protein-dependent manner [22]. These findings establish the relevance of cross-biofluid relationships but also raise further questions about their clinical interpretation. In particular, it remains important to distinguish whether associations between relative CSF–plasma signals and AD clinical stage reflect changes in CSF, plasma, or their differing disease-related patterns. An additional question is whether cross-sectionally identified analyte patterns are associated with subsequent clinical outcomes among individuals with mild cognitive impairment (MCI).

We therefore used paired CSF and plasma NULISAseq measurements from the Alzheimer’s Disease Neuroimaging Initiative to characterize analyte-specific cross-biofluid relationships and their associations with AD clinical stage. We compared corresponding CSF-only, plasma-only, and paired-difference (ΔNPQ) representations to distinguish their respective clinical-stage associations and identify analytes exhibiting different patterns across the two fluids. We subsequently examined whether a cross-sectionally derived multianalyte ΔNPQ pattern was associated with subsequent conversion from MCI to AD dementia. Finally, matched CSF-only, plasma-only, ΔNPQ, and joint CSF–plasma models were compared to evaluate the prognostic relevance of these representations in relation to established clinical and pathological biomarkers.

## 2. Methods

### 2.1. Study Cohort and Paired-Biofluid Profiling

**Figure 1:**
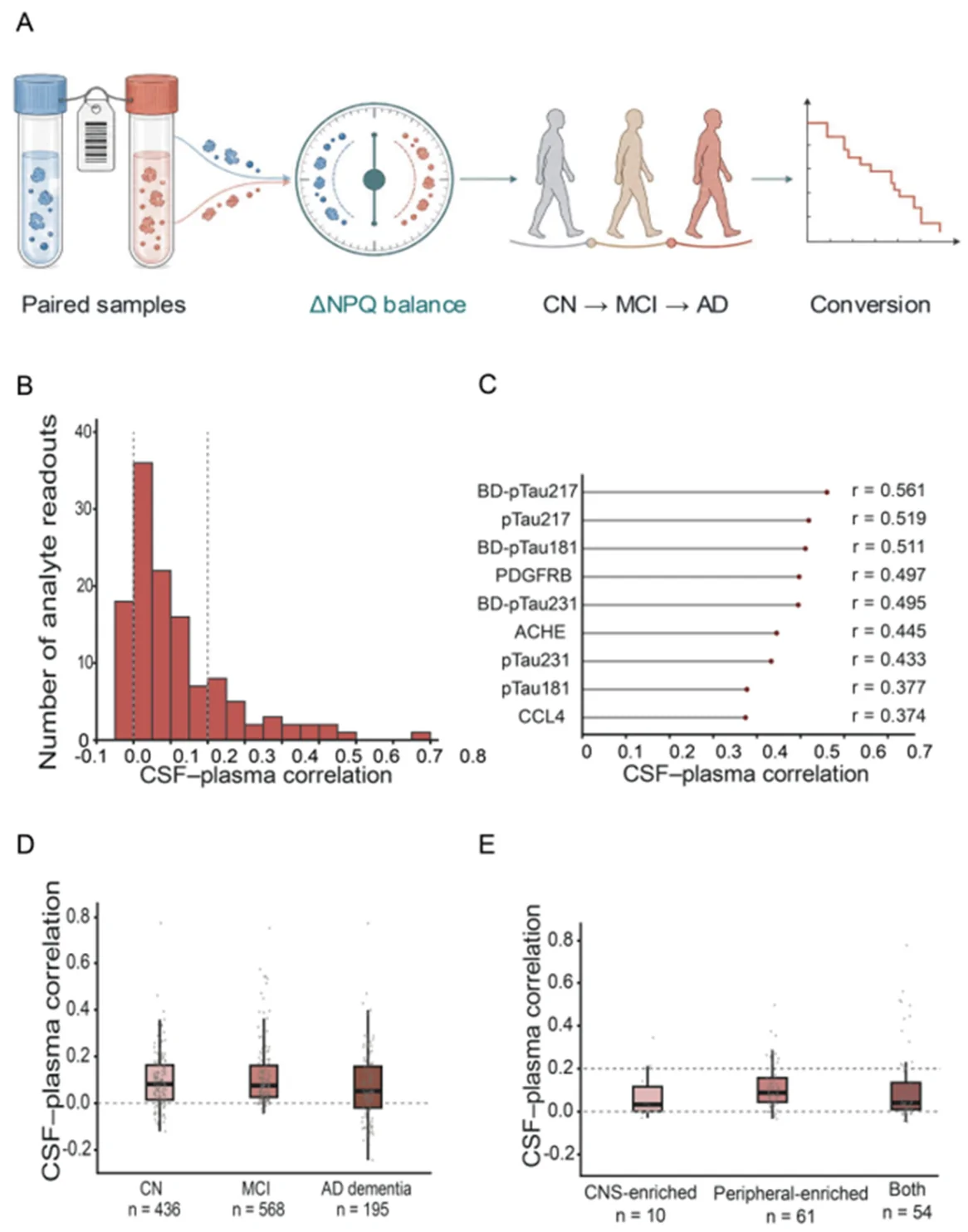
CSF–plasma coupling is heterogeneous across analyte readouts. (**A**) Paired-biofluid illustration linking matched CSF and plasma analyte signals to ΔNPQ relative balance, cross-sectional clinical stages, and MCI-to-AD dementia conversion. (**B**) Pearson correlation distribution between CSF and plasma NPQ values among 125 matched analyte readings. (**C**) CSF–plasma analyte readouts with highest correlation, sorted by Pearson correlation coefficient. (**D**) Correlation distribution stratified by diagnostic group. (**E**) Correlation stratified by GTEx-based source annotation. Vertical dashed lines indicate r = 0 and r = 0.20.

The data used in this study were obtained from the Alzheimer’s Disease Neuroimaging Initiative (ADNI) database. ADNI is a longitudinal, multicenter, public–private partnership established to develop and validate imaging, fluid, genetic, and clinical biomarkers of AD. Paired CSF and plasma NULISA proteomic data, together with demographic and clinical variables, were downloaded from the LONI Image and Data Archive on 5 May 2026. The present analysis used paired CSF and plasma measurements from the NULISAseq 120-plex CNS Disease Panel. Participants were included when matched CSF and plasma samples were collected within 30 days and clinical diagnostic information was available. The final paired cohort comprised 1199 participants (CN, n = 436; MCI, n = 568; AD dementia, n = 195) and 125 matched analyte readouts. Cross-sectional analyses used the full paired cohort. Longitudinal analyses used 547 participants with baseline MCI and follow-up diagnostic information, of whom 203 converted to AD dementia; non-converters were censored at their last available follow-up (Figure 1A).

### 2.2. Paired ΔNPQ Definition

Paired CSF and plasma proteins were measured using the NULISAseq CNS Disease Panel 120 on the automated Alamar ARGO™ HT platform. Analyses used the released internal-control/inter-plate-control-normalized NULISA Protein Quantification (NPQ) values without additional intensity normalization or post-release transformation. Detailed detection methods are provided in the Supplementary Materials. For each matched analyte and participant, paired ΔNPQ was defined as CSF NPQ minus plasma NPQ [22]. We defined ΔNPQ as an operational contrast between processed assay signals rather than an absolute concentration ratio or a direct measure of physiological protein distribution, transport, blood–brain barrier permeability, clearance, or tissue-specific production.

### 2.3. Statistical Analyses

Statistical analyses were performed using R version 4.4.2. Continuous variables are presented as the mean (SD) or median (IQR), and categorical variables as n (%). CSF–plasma coupling was assessed using Pearson correlations and Spearman analysis as a sensitivity analysis. Cross-sectional associations with clinical stage were examined using covariate-adjusted linear models with diagnosis coded ordinally as CN = 0, MCI = 1, and AD dementia = 2, adjusting for age, sex, and CSF and plasma assay plates; pairwise diagnostic contrasts and parallel CSF-only, plasma-only, and ΔNPQ models were additionally examined. Sensitivity analyses included additional adjustment for education and APOE ε4 status, exclusion of APP/MAPT-mapped readouts, resampling and plate-omission analyses, and an assessment of sex-related heterogeneity. Amyloid burden was defined as the inverse-standardized CSF Aβ42/40 ratio: amyloid burden = −1 × Z (CSF Aβ42/40 ratio). Accordingly, higher values of this derived measure correspond to lower CSF Aβ42/40 ratios and greater amyloid-related pathological burden. To quantify statistical overlap with established AD biomarkers, additional nested linear models evaluated the incremental and partial R^2^ attributable to CSF Aβ42/40-derived amyloid burden and CSF pTau217 for individual ΔNPQ readouts and the six-analyte score, adjusting for age, sex, and assay plates. Longitudinal analyses were conducted in participants with baseline MCI using matched six-analyte CSF-only, plasma-only, and ΔNPQ scores derived from the cross-sectional stage-associated pattern without using conversion outcomes for analyte selection, direction, or weighting. The same six standardized, direction-aligned analytes were equally weighted across the three representations to ensure comparability and avoid outcome-derived weight optimization; equal weighting was not intended to imply equivalent biological importance across analytes. To assess potential information reuse and diagnostic-stage-related confounding, an additional sensitivity analysis derived a new ΔNPQ score exclusively from CN and AD dementia participants, excluding all MCI participants from analyte selection and score construction. The resulting score was evaluated in the non-overlapping MCI cohort using the same Cox regression models. Cox proportional-hazards models for conversion to AD dementia were adjusted for age, sex, education, APOE ε4 status, baseline MMSE, and CDR-SB, with additional models adjusting for CSF Aβ42/40-derived amyloid burden and CSF pTau217. CSF-only, plasma-only, ΔNPQ, and joint CSF–plasma representations were compared using likelihood-ratio tests, AIC, and optimism-corrected Harrell’s C-indices based on 1000 bootstrap resamples. Proportional-hazards assumptions and competing non-AD dementia events were evaluated in sensitivity analyses. Multiple testing was controlled using the Benjamini–Hochberg false-discovery-rate procedure, with q < 0.05 considered significant.

## 3. Results

### 3.1. Demographics and Clinical Characteristics

The final paired CSF–plasma NULISAseq cohort included 1199 participants: 436 CN, 568 MCI, and 195 AD dementia. Baseline demographic, clinical, and biomarker characteristics are summarized in Table 1. Modest group differences were observed in age, sex distribution, and education, whereas the CSF–plasma collection interval did not differ across the diagnostic groups. APOE ε4 carrier frequency increased across the clinical groups, from 30.7% in CN to 50.0% in MCI and 71.3% in AD dementia (*p* < 0.001). Cognitive and functional measures followed the expected clinical gradient: the mean MMSE decreased from 29.1 in CN to 27.7 in MCI and 23.2 in AD dementia, while the median CDR-SB increased from 0.0 to 1.5 and 4.5 (both *p* < 0.001). AD-related fluid biomarkers also tracked clinical status. CSF Aβ42/40-derived amyloid burden increased across CN, MCI, and AD dementia, and CSF pTau217 increased in parallel (both *p* < 0.001).

### 3.2. CSF–Plasma Coupling Is Heterogeneous Across Analyte Readouts

A simple processing flow for our study is shown in Figure 1A. We quantified the correspondence between matched CSF and plasma NPQ values across the 125 shared analyte readouts. Correlations were positive for 107 readouts but were generally modest: the median Pearson correlation was 0.071, 26 readouts exceeded r = 0.20, and only four exceeded r = 0.50 (Figure 1B). The results of the sensitivity analysis are shown in Supplementary Table S1.

Coupling was strongly analyte-dependent. MAPT-mapped readouts showed the highest correlations, followed by selected vascular, cholinergic, and inflammatory readouts including PDGFRB, ACHE, and CCL4 (Figure 1C). The median coupling was 0.082 in CN, 0.076 in MCI, and 0.052 in AD dementia (Figure 1D), indicating that the two biofluids cannot be treated as interchangeable measurement spaces.

GTEx-based source annotation did not explain this heterogeneity (Supplementary Method). The proportion of readouts exceeding r = 0.20 was 20% among CNS-enriched targets, 20% among peripheral-enriched targets, and 22% among targets expressed in both categories (Figure 1E). The analyte-specific coupling landscape therefore provided the empirical basis for testing relative CSF–plasma balance directly.

### 3.3. Fourteen ΔNPQ Readouts Are Associated with Clinical Stage

**Figure 2:**
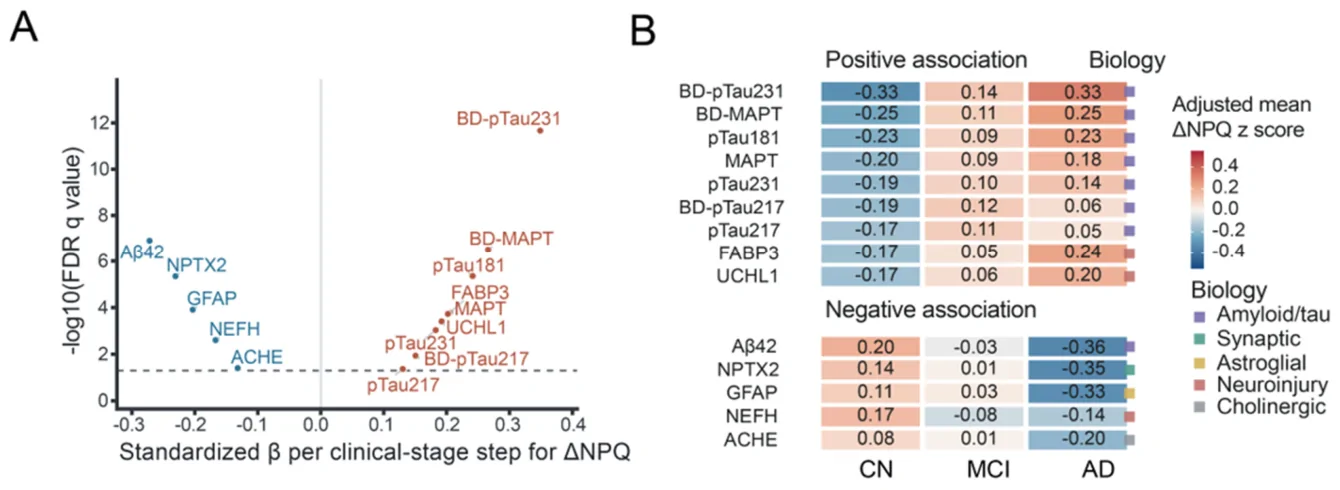
Fourteen ΔNPQ readouts are associated with clinical stage. (**A**) A volcano plot of standardized ΔNPQ associations with ordinal clinical stage across 125 paired analyte readouts. The horizontal dashed line indicates FDR q = 0.05. (**B**) Model-adjusted mean standardized ΔNPQ values across CN, MCI, and AD dementia for the 14 FDR-significant readouts. The color intensity represents adjusted mean ΔNPQ z scores. Diagnostic groups are cross-sectional and do not represent within-person progression.

We tested each of the 125 ΔNPQ readouts against ordinal clinical stage, with adjustment for age, sex, and CSF/plasma assay plates (Supplementary Table S1). Fourteen readouts were associated with more advanced stage after FDR correction (Figure 2A). These readouts mapped to eight gene-level targets, defining a compact cross-biofluid pattern rather than 14 independent protein discoveries.

The pattern was bidirectional and biologically structured (Table 2). Seven MAPT-mapped readouts, FABP3, and UCHL1 showed higher ΔNPQ with more advanced stage, whereas Aβ42, NPTX2, GFAP, NEFH, and ACHE showed lower ΔNPQ. Thus, the stage-associated pattern extended from canonical amyloid/tau biology to synaptic, astroglial, cholinergic, and neuroaxonal signals.

Adjusted group summaries showed coherent stage ordering for the strongest readouts, with MCI generally occupying an intermediate position between CN and AD dementia (Figure 2B). Other readouts showed stage-restricted differences, indicating that cross-biofluid remodeling was coordinated but not uniform across the clinical spectrum.

We therefore defined these 14 readouts as the core clinical-stage-associated ΔNPQ pattern and carried them forward into stage-contrast, matched single-fluid decomposition, robustness, cognitive-context, healthy-aging, and longitudinal analyses.

### 3.4. Distinct CSF and Plasma Contributions Shape ΔNPQ Remodeling

**Figure 3:**
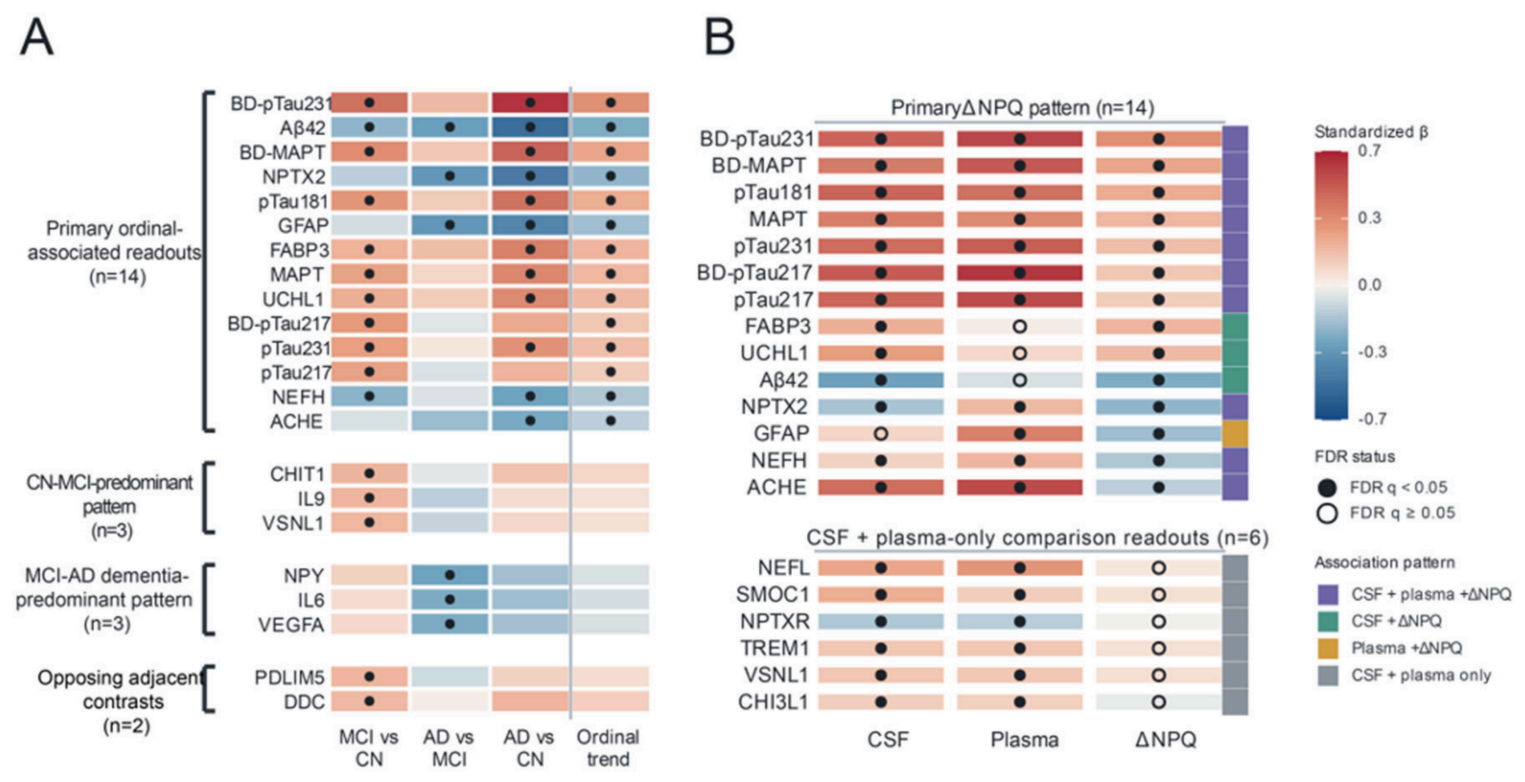
Distinct CSF and plasma contributions shape ΔNPQ remodeling. (**A**) Adjusted pairwise ΔNPQ contrasts for MCI versus CN, AD dementia versus MCI, and AD dementia versus CN among the 14 core readouts. (**B**) Parallel CSF-only, plasma-only, and ΔNPQ clinical-stage associations for the 14 primary ΔNPQ readouts and selected comparison readouts associated with clinical stage in both single-biofluid models but not in the ΔNPQ model.

Categorical contrasts separated ordered from stage-restricted components of the core pattern. MAPT-mapped readouts, FABP3, and UCHL1 showed broadly ordered differences across successively more advanced groups, whereas Aβ42, NPTX2, GFAP, NEFH, and ACHE shifted in the opposite direction (Figure 3A). Selected immune, vascular, and neuronal-signaling readouts were concentrated in either the CN-to-MCI or MCI-to-AD dementia contrast. These contrasts show that the dominant ΔNPQ pattern spans the clinical spectrum while a smaller set of analytes marks specific cross-sectional transitions. The result is an ordered molecular backbone with context-dependent remodeling around it.

We then decomposed the same clinical-stage associations into matched CSF-only, plasma-only, and ΔNPQ representations (Figure 3B; Supplementary Table S2). This analysis distinguished changes shared across the two fluids from shifts in their relative balance.

Aβ42, FABP3, and UCHL1 were associated with stage in CSF and ΔNPQ but not plasma, whereas GFAP was associated in plasma and ΔNPQ but not CSF at FDR significance. MAPT-mapped readouts, NPTX2, NEFH, and ACHE were associated across CSF, plasma, and ΔNPQ. This heterogeneity shows that the core pattern is generated by different configurations of matched biofluid change rather than one uniform compartmental response. Conversely, NEFL, SMOC1, NPTXR, TREM1, VSNL1, and CHI3L1 were associated with stage in both single-biofluid models without a significant ΔNPQ association. Thus, no statistically significant stage-related difference in their paired signal contrasts was detected.

### 3.5. The Core ΔNPQ Pattern Shows Internal Stability and Broader Clinical Context

The core ΔNPQ pattern remained directionally stable across covariate, resampling, plate-omission, and sex-related sensitivity analyses (Supplementary Figure S1). Secondary analyses of MMSE and healthy aging provided limited evidence beyond the clinical-stage associations (Supplementary Figure S2); detailed results are provided in the Supplementary Materials.

### 3.6. Longitudinal Associations of Matched Biofluid Representations with MCI Conversion

**Figure 4:**
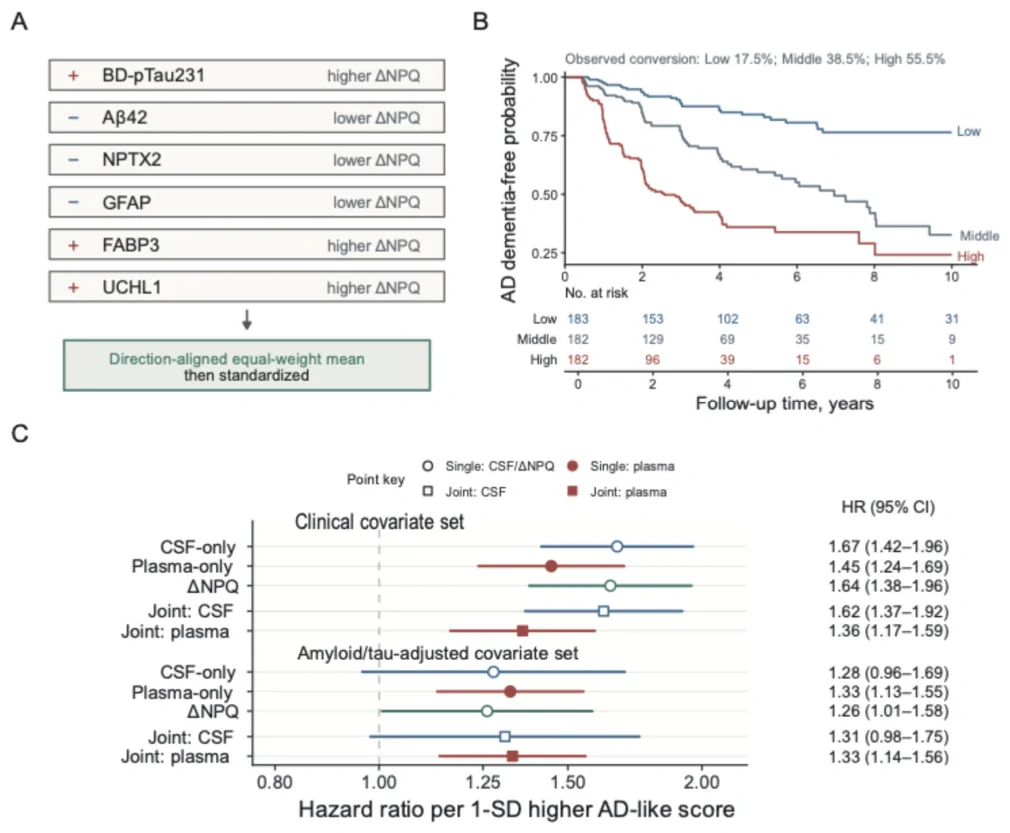
Longitudinal associations of matched biofluid representations with conversion from MCI to AD dementia. (**A**) Construction of the cross-sectionally derived, six-analyte score constructed without using conversion outcomes. Components were direction-aligned, equally weighted, and not selected, directed, or weighted using conversion outcomes. (**B**) Kaplan–Meier curves for AD dementia-free probability among baseline MCI participants stratified by ΔNPQ score tertiles. Numbers at risk are shown below the plot. (**C**) Cox-model associations for matched CSF-only, plasma-only, ΔNPQ, and joint CSF–plasma score representations under clinical and amyloid/tau-adjusted covariate sets. Hazard ratios are shown per 1-SD higher AD-like score.

The longitudinal cohort included 547 participants with baseline MCI, of whom 203 converted to Alzheimer’s disease dementia during a median follow-up of 3.0 years (IQR, 1.6–5.3 years; Supplementary Table S3). We constructed matched six-analyte CSF-only, plasma-only, and ΔNPQ scores using BD-pTau231, Aβ42, NPTX2, GFAP, FABP3, and UCHL1 (Figure 4A). Components were direction-aligned to the corresponding cross-sectional stage model and equally weighted without using conversion outcomes. ΔNPQ score tertiles separated the cohort into observed conversion rates of 17.5%, 38.5%, and 55.5% from low to high score (Figure 4B).

In models adjusted for demographic, genetic, and baseline clinical variables, each single-score representation was associated with conversion (Figure 4C; Table 3). The HR per 1-SD higher score was 1.67 (95% CI, 1.42–1.96) for CSF-only, 1.45 (95% CI, 1.24–1.69) for plasma-only, and 1.64 (95% CI, 1.38–1.96) for ΔNPQ. Their optimism-corrected increases in C-index values were 0.0247, 0.0163, and 0.0207, respectively.

Joint inclusion of CSF-only and plasma-only scores produced the strongest overall model. Both components remained independently associated with conversion, the optimism-corrected C-index reached 0.7980, and discrimination increased by 0.0347 (95% CI, 0.0182–0.0532) over that for the clinical base model. The joint model also had the lowest AIC. Thus, the ΔNPQ score summarized substantial paired-biofluid information in a single representation, whereas separate modeling of the CSF-only and plasma-only scores retained more complete prognostic information.

Direct pairwise comparisons provided no clear evidence that ΔNPQ outperformed either matched single-fluid representation. In the clinical model, the optimism-corrected C-index difference was −0.0040 for ΔNPQ versus CSF-only (95% CI, −0.0156 to 0.0062), 0.0044 for ΔNPQ versus plasma-only (95% CI, −0.0171 to 0.0233), and −0.0140 for ΔNPQ versus the joint representation (95% CI, −0.0278 to 0.0008). Among the remaining comparisons, the joint model showed higher discrimination than the plasma-only model, whereas its difference from the CSF-only model was not clearly supported. After amyloid/tau adjustment, none of the pairwise comparisons provided clear evidence of a difference in discrimination (Supplementary Table S4).

After adjustment for CSF Aβ42/40-derived amyloid burden and CSF pTau217, the CSF-only score was attenuated (HR, 1.28; 95% CI, 0.96–1.69), whereas ΔNPQ retained a modest association (HR, 1.26; 95% CI, 1.01–1.58) but added little discrimination (corrected ΔC-index, 0.0023; 95% CI, −0.0006 to 0.0118). Plasma-only remained associated with conversion (HR, 1.33; 95% CI, 1.13–1.55) and improved discrimination, while the joint model retained the largest residual gain. These findings indicate limited incremental prognostic value of ΔNPQ beyond that of established CSF amyloid/tau biomarkers.

Complementary variance-partitioning analyses showed that CSF amyloid burden and pTau217 accounted for 49.2% of the residual variance in the six-analyte ΔNPQ score after adjustment for age, sex, and assay plates (partial R^2^ = 0.492), with a similar estimate in the MCI cohort (partial R^2^ = 0.490). However, the degree of statistical overlap varied substantially across individual readouts, with relatively low partial R^2^ values for NPTX2, GFAP, and NEFH (Supplementary Table S6).

Additional time-to-event sensitivity analyses were consistent with the main Cox findings. The information remaining beyond core CSF amyloid/tau measures was therefore expressed most clearly in plasma.

Sensitivity analyses supported this interpretation. ΔNPQ score-specific proportional-hazards tests were not significant, and age-by-log(time) models produced similar score estimates. Fine–Gray models treating nine non-AD dementia events as competing events were directionally consistent, with ΔNPQ strongly associated in the clinical model and borderline after amyloid/tau adjustment, while plasma-only remained significant after amyloid/tau adjustment.

In an additional sensitivity analysis excluding all MCI participants from the discovery cohort, a 10-analyte ΔNPQ score derived from CN and AD dementia participants remained associated with subsequent MCI conversion after adjustment for baseline clinical characteristics (HR, 1.46; 95% CI, 1.22–1.75; *p* < 0.001; optimism-corrected ΔC-index, 0.0122). However, the association was attenuated after further adjustment for amyloid/tau biomarkers (HR, 1.17; 95% CI, 0.96–1.43; *p* = 0.114; optimism-corrected ΔC-index, 0.0006; Supplementary Table S7).

## 4. Discussion

In this study, we characterized analyte-specific relationships between matched CSF and plasma signals across the AD clinical spectrum and examined their relevance to subsequent dementia conversion in MCI. CSF–plasma coupling was generally weak and heterogeneous across analytes, indicating that corresponding molecular signals were not consistently represented across the two biofluids. Clinical-stage analyses identified 14 analyte readouts mapping to eight gene-level targets with distinct directions of relative CSF–plasma association. Importantly, matched single-fluid analyses showed that some stage-associated signals were evident in both fluids without corresponding changes in ΔNPQ, whereas others exhibited differences in their relative cross-biofluid signals. These findings extend previous paired-proteomic observations of protein-specific CSF–plasma relationships in aging and cognitive impairment [22] by characterizing their associations with AD clinical stage and examining the subsequent clinical relevance of selected analyte patterns.

The biological composition of the stage-associated pattern suggests that analyte-specific cross-biofluid heterogeneity extends beyond canonical amyloid and tau biomarkers. In addition to APP- and MAPT-mapped readouts, the identified analytes included NPTX2, GFAP, FABP3, UCHL1, NEFH, and ACHE, representing molecular processes related to synaptic function, astroglial responses, neuronal integrity, metabolism, and cholinergic signaling. These readouts should not be interpreted as a single coordinated molecular pathway, particularly because multiple tau-related measurements represent different assay readouts of the same gene-level target. Instead, their heterogeneous cross-biofluid patterns highlight the importance of considering analyte-specific relationships when interpreting disease-associated molecular signals. Previous CSF proteomic studies have identified synaptic signatures, including NPTX2-related measures, associated with cognitive resilience and decline beyond established amyloid and tau pathology [23]. Conversely, plasma GFAP has shown stronger associations with amyloid pathology and subsequent clinical progression than its corresponding CSF measurement in some cohorts [24]. Experimental evidence from a 6-OHDA rat model has also demonstrated rapid local amyloid-β accumulation following nigral injury, including amyloid-β signals in and around cerebral blood vessels [25]. Although these observations do not directly establish mechanisms underlying paired CSF–plasma analyte differences in AD, they highlight the potential influence of local tissue pathology on amyloid-related molecular signals. These biological annotations provide context for interpreting the identified analytes but do not establish the physiological mechanisms underlying their observed cross-biofluid signal patterns. Rather, our findings indicate that AD-related analytes exhibit heterogeneous clinical-stage associations across CSF and plasma, without directly demonstrating changes in protein distribution or intercompartmental transport.

Age-related changes may contribute to the observed ΔNPQ patterns, particularly for NEFH, BD-MAPT, and BD-pTau231, which showed overlapping associations with age and clinical stage. However, only three of the 14 stage-associated readouts overlapped with age-associated readouts in cognitively normal participants and in the primary stage models adjusted for age, suggesting that chronological aging alone is unlikely to explain the broader pattern. Nevertheless, residual aging-related influences cannot be excluded, as normative aging and preclinical AD-related processes may overlap in this cross-sectional cohort.

The longitudinal analysis further linked these cross-biofluid patterns to clinical heterogeneity within MCI. Individuals with MCI exhibit variable subsequent clinical trajectories, consistent with previous observations of heterogeneous neurodegenerative patterns and their relationships with cognitive outcomes [26]. In the present study, a six-analyte ΔNPQ score derived from cross-sectional clinical-stage associations was associated with later conversion to AD dementia after adjustment for demographic, genetic, and baseline clinical characteristics. Although score components were selected and direction-aligned without using conversion outcomes, the original discovery and prognostic cohorts overlapped. The non-overlapping discovery sensitivity analysis supported the association after adjustment for baseline clinical characteristics, although attenuation following amyloid/tau adjustment suggests that the prognostic information partly overlaps with established AD pathology. Variance-partitioning analyses further revealed substantial statistical overlap between the six-analyte ΔNPQ score and established amyloid/tau biomarkers (partial R^2^ = 0.492), although this overlap varied across individual readouts. These findings support interpreting ΔNPQ primarily as a measure of analyte-specific cross-biofluid associations rather than as evidence of independent prognostic utility or distinct biological mechanisms. Nevertheless, association with conversion should be distinguished from incremental prognostic utility. Direct comparisons did not establish a discrimination advantage of ΔNPQ over matched single-biofluid representations, while joint inclusion of CSF and plasma scores yielded the largest estimated improvement in discrimination. Additional adjustment for CSF amyloid/tau biomarkers attenuated the ΔNPQ association and left little incremental discrimination, whereas the plasma-only representation retained a clearer residual association with conversion. Thus, the longitudinal findings support the clinical relevance of selected cross-biofluid patterns but do not establish ΔNPQ as a superior prognostic representation or an independent predictor beyond established AD biomarkers.

These observations also have methodological implications for the interpretation of paired-biofluid biomarker studies. Conventional approaches frequently assess whether plasma measurements reproduce associations established in CSF, yet previous studies have reported limited cross-fluid correspondence despite clinically relevant associations within individual compartments [27]. More recent paired-proteomic analyses have demonstrated that CSF–plasma protein relationships vary across analytes and disease-related conditions, while other investigations have identified differing associations of CSF and plasma proteomic profiles with APOE ε4 status, amyloid pathology, and cognitive decline [22,28]. Our matched single-fluid decomposition highlights two related but distinct questions: whether an analyte is associated with clinical stage within CSF or plasma, and whether its relative signal differs between the two fluids. The ΔNPQ representation summarizes the latter relationship in a compact form, but it does not constitute an independent molecular measurement. Because the underlying CSF and plasma NPQ values were normalized within separate assay matrices, differences between them cannot be interpreted as absolute concentration ratios, direct evidence of protein redistribution, or measures of blood–brain barrier transport and clearance. Moreover, ΔNPQ is mathematically derived from its constituent measurements and cannot be assumed to provide additional predictive information beyond a model retaining both source signals separately. Its principal value therefore lies in characterizing relative cross-biofluid patterns rather than demonstrating inherent predictive superiority.

Several limitations should be considered. First, this study was conducted exclusively within ADNI, which may limit the generalizability of our findings to more heterogeneous clinical populations. Future studies should replicate the identified cross-biofluid analyte patterns and evaluate the prespecified score in independent, multicenter memory-clinic cohorts with broader demographic characteristics and comorbidity profiles, using harmonized paired-biofluid measurements and assessing prognostic performance beyond established AD biomarkers. Second, although conversion outcomes were not used to select or weight the original six-analyte score, the longitudinal MCI cohort overlapped with the clinical-stage discovery sample. While the non-overlapping discovery sensitivity analysis addressed this potential information reuse, residual confounding by baseline disease severity cannot be entirely excluded. Third, matrix-specific normalization, separate assay processing, and residual technical variability may influence the interpretation of ΔNPQ, even after adjustment for assay-plate effects. Sex-related differences in astroglial responses, neuroinflammatory processes, and peripheral biomarker regulation provide biological plausibility for sex-dependent analyte profiles. Although no sex-by-diagnosis interaction survived FDR correction, the smaller sex-specific subgroups, particularly within the AD dementia group, and multiple-testing correction limited sensitivity to detect modest interaction effects; therefore, the absence of significant interactions should not be interpreted as evidence of equivalent associations between sexes. Finally, conversion to AD dementia was clinically defined and did not necessarily represent biomarker-confirmed AD etiology. Future longitudinal studies with repeated paired CSF and plasma sampling are also needed to determine whether these analyte-specific signal patterns change within individuals over time.

In conclusion, paired CSF–plasma analysis identified analyte-specific molecular patterns associated with AD clinical stage, while a cross-sectionally derived multianalyte representation was also associated with subsequent dementia conversion in MCI. These findings support the characterization of cross-biofluid molecular heterogeneity as a complementary perspective on AD-associated biomarker variation, rather than a demonstrably superior prognostic approach. Further longitudinal and external validation will be necessary to establish its biological interpretation and potential clinical utility.

## Figures and Tables

**Table 1:** Baseline characteristics of participants with paired CSF–plasma.

Variable	Overall	CN	MCI	AD Dementia	*p*
Participants, n	1199	436	568	195	
Age, years	72.7 (7.2)	72.6 (6.2)	72.3 (7.6)	74.3 (8.1)	0.002
Female sex, n (%)	568 (47.4%)	256 (58.7%)	233 (41.0%)	79 (40.5%)	<0.001
Education, years	16.2 (2.7)	16.5 (2.5)	16.1 (2.7)	15.5 (3.0)	<0.001
APOE ε4 carrier, n (%)	557 (46.5%)	134 (30.7%)	284 (50.0%)	139 (71.3%)	<0.001
MMSE	27.5 (2.6)	29.1 (1.1)	27.7 (1.9)	23.2 (2.0)	<0.001
CDR-SB	1.0 [0.0, 2.0]	0.0 [0.0, 0.0]	1.5 [1.0, 2.0]	4.5 [3.5, 5.0]	<0.001
CSF Aβ42/40-derived amyloid burden, z score	0.00 (1.00)	−0.34 (0.93)	0.11 (0.99)	0.42 (0.93)	<0.001
CSF pTau217, NPQ	15.44 (1.42)	14.88 (1.22)	15.59 (1.45)	16.28 (1.25)	<0.001
CSF–plasma collection interval, days	0 [0, 3]	0 [0, 1]	0 [0, 5]	0 [0, 4]	0.367

Values are the mean (SD), median [IQR], or n (%) as indicated. *p* values are descriptive and were not used for primary inference. *p* values were calculated using one-way ANOVA for mean (SD) variables, Kruskal–Wallis tests for median [IQR] variables, and Pearson χ^2^ tests for categorical variables. APOE ε4 carrier status was defined as the presence of at least one ε4 allele based on ADNI genotype data. Amyloid burden z score was calculated as −1 × z (CSF Aβ42/40 ratio), so higher values indicate lower CSF Aβ42/40 and greater amyloid pathology. CSF pTau217 is reported in NPQ units. CDR-SB was available for 1183 of 1199 participants (CN, 426/436; MCI, 564/568; AD dementia, 193/195) and was taken from the nearest available assessment within 180 days before paired sampling.

**Table 2:** Clinical-stage-associated CSF–plasma ΔNPQ readouts.

Protein	Biological Category	Standardized β per AD Continuum Step	95% CI	*p* Value	FDR q Value
BD-pTau231	Amyloid/tau	0.349	0.261 to 0.437	<0.001	<0.001
Aβ42	Amyloid/tau	−0.272	−0.360 to −0.184	<0.001	<0.001
BD-MAPT	Amyloid/tau	0.266	0.176 to 0.356	<0.001	<0.001
pTau181	Amyloid/tau	0.241	0.152 to 0.331	<0.001	<0.001
NPTX2	Synaptic	−0.231	−0.317 to −0.145	<0.001	<0.001
GFAP	Astroglial	−0.203	−0.291 to −0.116	<0.001	<0.001
FABP3	Neuroinjury-related	0.205	0.115 to 0.296	<0.001	<0.001
MAPT	Amyloid/tau	0.202	0.112 to 0.292	<0.001	<0.001
UCHL1	Neuroinjury-related	0.192	0.102 to 0.282	<0.001	<0.001
pTau231	Amyloid/tau	0.183	0.093 to 0.273	<0.001	<0.001
NEFH	Neuroinjury-related	−0.167	−0.255 to −0.079	<0.001	0.002
BD-pTau217	Amyloid/tau	0.151	0.060 to 0.241	0.001	0.012
ACHE	Cholinergic	−0.132	−0.222 to −0.042	0.004	0.039
pTau217	Amyloid/tau	0.130	0.040 to 0.221	0.005	0.043

Values were obtained from covariate-adjusted linear models of standardized ΔNPQ with cross-sectional clinical stage coded as CN = 0, MCI = 1, and Alzheimer’s disease dementia = 2. Standardized β represents the change in ΔNPQ per one-category increase in clinical stage and does not represent within-person disease progression. The 14 assay readouts correspond to eight gene-level targets. Seven readouts map to MAPT, Aβ42 maps to APP, and the remaining readouts map to NPTX2, GFAP, FABP3, UCHL1, NEFH, and ACHE. FDR q values were calculated using the Benjamini–Hochberg procedure.

**Table 3:** Matched comparison of six-analyte biofluid score models for MCI-to-AD dementia conversion.

Covariate Set	Added Representation	HR per 1-SD Higher Score (95% CI)	*p* Value	Optimism-Corrected C-Index	Corrected ΔC-Index vs. Base (95% CI)	ΔAIC	LRT *p*
Clinical	Base	—	—	0.7634	—	0.00	—
	CSF-only score	1.67 (1.42–1.96)	<0.001	0.7880	0.0247 (0.0105–0.0429)	−35.69	<0.001
	Plasma-only score	1.45 (1.24–1.69)	<0.001	0.7797	0.0163 (0.0058–0.0317)	−19.35	<0.001
	ΔNPQ score	1.64 (1.38–1.96)	<0.001	0.7841	0.0207 (0.0072–0.0410)	−29.80	<0.001
	Joint CSF + plasma scores	CSF: 1.62 (1.37–1.92) Plasma: 1.36 (1.17–1.59)	CSF: <0.001 Plasma: <0.001	0.7980	0.0347 (0.0182–0.0532)	−48.76	<0.001
Amyloid/tau-adjusted	Base	—	—	0.7837	—	0.00	—
	CSF-only score	1.28 (0.96–1.69)	0.087	0.7873	0.0037 (−0.0004–0.0124)	−0.92	0.087
	Plasma-only score	1.33 (1.13–1.55)	<0.001	0.7918	0.0082 (0.0014–0.0200)	−10.34	<0.001
	ΔNPQ score	1.26 (1.01–1.58)	0.044	0.7860	0.0023 (−0.0006–0.0118)	−2.09	0.043
	Joint CSF + plasma scores	CSF: 1.31 (0.98–1.75) Plasma: 1.33 (1.14–1.56)	CSF: 0.066 Plasma: <0.001	0.7954	0.0117 (0.0037–0.0264)	−11.71	<0.001

The CSF-only, plasma-only, and ΔNPQ scores were constructed from the same six analytes, aligned to the corresponding cross-sectional clinical-stage direction, equally weighted, and standardized before Cox modeling. Hazard ratios are reported per 1-SD higher stage-aligned score. The clinical base model included age, sex, education, APOE ε4 carrier status, baseline MMSE, and baseline CDR-SB. The amyloid/tau-adjusted model additionally included CSF Aβ42/40-derived amyloid burden and CSF pTau217. C-indices were corrected for optimism using 1000 participant-level bootstrap samples. Corrected ΔC-index represents the change relative to the corresponding base model. The joint representation included both the CSF-only and plasma-only scores.

## Data Availability

The datasets analyzed during the current study are available through the Alzheimer’s Disease Neuroimaging Initiative database, subject to approval and compliance with the ADNI Data Use Agreement. The derived results supporting the findings of this study are included in the article and its Supplementary Information files.

## Supplementary Materials

The following supporting information can be downloaded at: https://www.mdpi.com/article/10.3390/biomedicines14102407/s1.
