# Supplementary material for: Paired CSF–Plasma Analyte Signal Contrasts Across Alzheimer’s Disease Clinical Stages and MCI-to-Dementia Conversion
Source: Biomedicines. 2026 Oct 21;14(10):2407. doi: 10.3390/biomedicines14102407 (PMC13701971; doi:10.3390/biomedicines14102407)
Supplement: Supplementary file 1 [file biomedicines-14-02407-s001.zip › biomedicines-4645939-supplementary.pdf]

Paired CSF–Plasma Analyte Signal Contrasts Across Alzheimer's Disease Clinical Stages and MCI-to-Dementia Conversion

Yuepeng Deng [1,2,†], Dandan Chen [1,3,†], Haolin Yin [1,2], Zihao Lu [2], Xiaohe Tian [1,2,*] and Qiyong Gong [1,2,*] on behalf of the Alzheimer's Disease Neuroimaging Initiative (ADNI)

[1] Department of Radiology, Huaxi MR Research Center (HMRRC), Institute of Radiology and Medical Imaging, Psychoradiology Key Laboratory of Sichuan Province, West China Hospital of Sichuan University, Chengdu 610041, China

[2] Xiamen Key Lab of Psychoradiology and Neuromodulation, Department of Radiology, West China Xiamen Hospital of Sichuan University, Xiamen 361000, China

[3] Department of Radiology and National Clinical Research Center for Geriatrics, Huaxi MR Research Center (HMRRC), Frontiers Science Center for Disease-Related Molecular Network, West China Hospital of Sichuan University, Chengdu 610041, China

* Correspondence: xiaohe.t@wchscu.cn (X.T.); qiyonggong@hmrrc.org.cn (Q.G.)

† These authors contributed equally to this work.

Supplementary

Supplementary Methods

NULISAseq assay, normalization, and quality control

Paired CSF and plasma protein measurements were generated at the Banner Sun Health Research Institute using the NULISAseq CNS Disease Panel 120 on the automated Alamar ARGO™ HT platform. The overall project assayed 1,428 plasma samples and 1,633 CSF samples across 37 assay plates. Samples were stored at -80°C until analysis, thawed, and centrifuged at 2,200 × *g* for 10 min, after which 25 µL of supernatant was transferred to a 96-well plate for analysis. Each assay plate included three pooled-plasma sample-control replicates, three independent pooled-plasma inter-plate control (IPC) replicates, and four negative controls. An exogenous internal-control (IC) reporter was added to every well for within-well normalization.

NULISA Protein Quantification (NPQ) values were generated using sequential IC and IPC normalization. For each target, raw counts were first divided by the IC count in the corresponding well. The resulting IC-normalized counts were then divided by the target-specific median across the three IPC replicates on the same plate, multiplied by 10^4, offset by +1, and log2-transformed to generate NPQ values. Thirty CSF bridge samples were included on the first plate of both the plasma and CSF assay series to support potential future cross-cohort harmonization. We analyzed the released IC/IPC-normalized NPQ values without additional bridge-sample correction, intensity normalization, or post-release transformation.

Limits of detection were defined separately for each target and assay plate using the negative-control distribution on the unlogged normalized-count scale and were subsequently rescaled and log2-transformed to the NPQ scale. Of the 3,061 samples processed in the overall project, 2,992 passed sample-level quality control and 69 received QC warnings. In accordance with the data-provider guidance, QC warnings were not treated as automatic exclusion criteria. Individual NPQ values below the limit of detection were retained without censoring or imputation, whereas paired analyses required nonmissing NPQ measurements in both CSF and plasma for the corresponding participant and analyte.

Because CSF and plasma samples were processed on separate assay plates, the corresponding CSF and plasma plate identifiers were retained separately and included as covariates in the primary statistical models. Leave-one-plate-out analyses were performed to evaluate the sensitivity of the main findings to residual plate-related effects.

GTEx-based expression annotation

To provide descriptive context, analyte targets were mapped to gene symbols and annotated using bulk-tissue RNA-seq data from GTEx[1]. Following published approaches[2, 3], a gene was classified as brain-enriched when its maximal expression across brain regions was at least fourfold higher than in any non-brain tissue, peripheral-enriched when its maximal non-brain expression was at least fourfold higher than maximal brain expression, and mixed otherwise. GTEx expression values were normalized using the DESeq2 R package[4]. These categories were used only as transcript-expression annotations. Transcript abundance does not establish the tissue origin of a measured CSF or plasma protein, and the categories were neither used to define the primary models nor interpreted as evidence of analyte production, release, transport or compartment dominance.

Supplementary Results

Internal stability and non-APP/MAPT sensitivity

The core 14-readout pattern remained directionally stable across complementary sensitivity analyses (Supplementary Figure S1A and Supplementary Table 6). After additional adjustment for education and APOE ε4 carrier status, all 14 readouts retained their original association direction; 11 remained FDR significant, whereas three no longer met the FDR threshold.

Predefined exclusion of APP- and MAPT-mapped assays retained five non-APP/MAPT clinical-stage-associated readouts: NPTX2, GFAP, FABP3, UCHL1 and NEFH (Supplementary Figure S1B). NPTX2, GFAP, FABP3 and NEFH remained FDR significant after additional adjustment for education and APOE ε4 carrier status, whereas UCHL1 retained a directionally consistent nominal

association. These findings indicate that the paired-biofluid pattern was not solely attributable to multiple APP- and MAPT-mapped assay readouts.

Resampling, plate and sex sensitivity analyses

Repeated stratified split-sample analyses and diagnosis-stratified bootstrap resampling supported internal directional consistency. BD-pTau231, Aβ42, BD-MAPT, NPTX2, pTau181, GFAP and FABP3 showed the strongest held-out directional support. All 14 core readouts retained their original association direction across bootstrap iterations, although the frequency of FDR-significant support varied across individual readouts.

Leave-one-plate-out analyses preserved association direction across CSF- and plasma-plate omission scenarios, with most core readouts remaining FDR significant in nearly all runs. Sex-stratified estimates were directionally concordant, and no core readout showed an FDR-significant sex-by-diagnosis interaction. These results support internal stability but do not substitute for validation in an independent cohort.

Cognitive and healthy-aging context

Thirteen ΔNPQ readouts were associated with MMSE after FDR correction in models adjusted for age, sex, education and assay plates (Supplementary Figure S2A and Supplementary Table 7). Their directions were concordant with the core clinical-stage pattern: higher MAPT-mapped, FABP3 and UCHL1 ΔNPQ values and lower Aβ42, NPTX2, GFAP, ACHE and NEFH ΔNPQ values were associated with worse cognition. CRH was associated with MMSE but was not part of the core clinical-stage pattern. After additional adjustment for diagnosis, no readout remained FDR significant, indicating that the MMSE associations largely reflected the cognitive gradient across diagnostic groups rather than diagnosis-independent cognitive effects.

In cognitively normal participants, 19 ΔNPQ readouts were associated with age after FDR correction (Supplementary Figure S2B and Supplementary Table 8). NEFH, BD-MAPT and BD-pTau231 showed FDR-significant associations in both the clinical-stage and cognitively normal age

models, whereas the remaining core readouts were significant only in the clinical-stage model. This limited FDR-significant overlap provides descriptive evidence that the clinical-stage pattern was not fully recapitulated by the detected healthy-aging associations. Formal differences between age- and clinical-stage-associated effects were not tested.

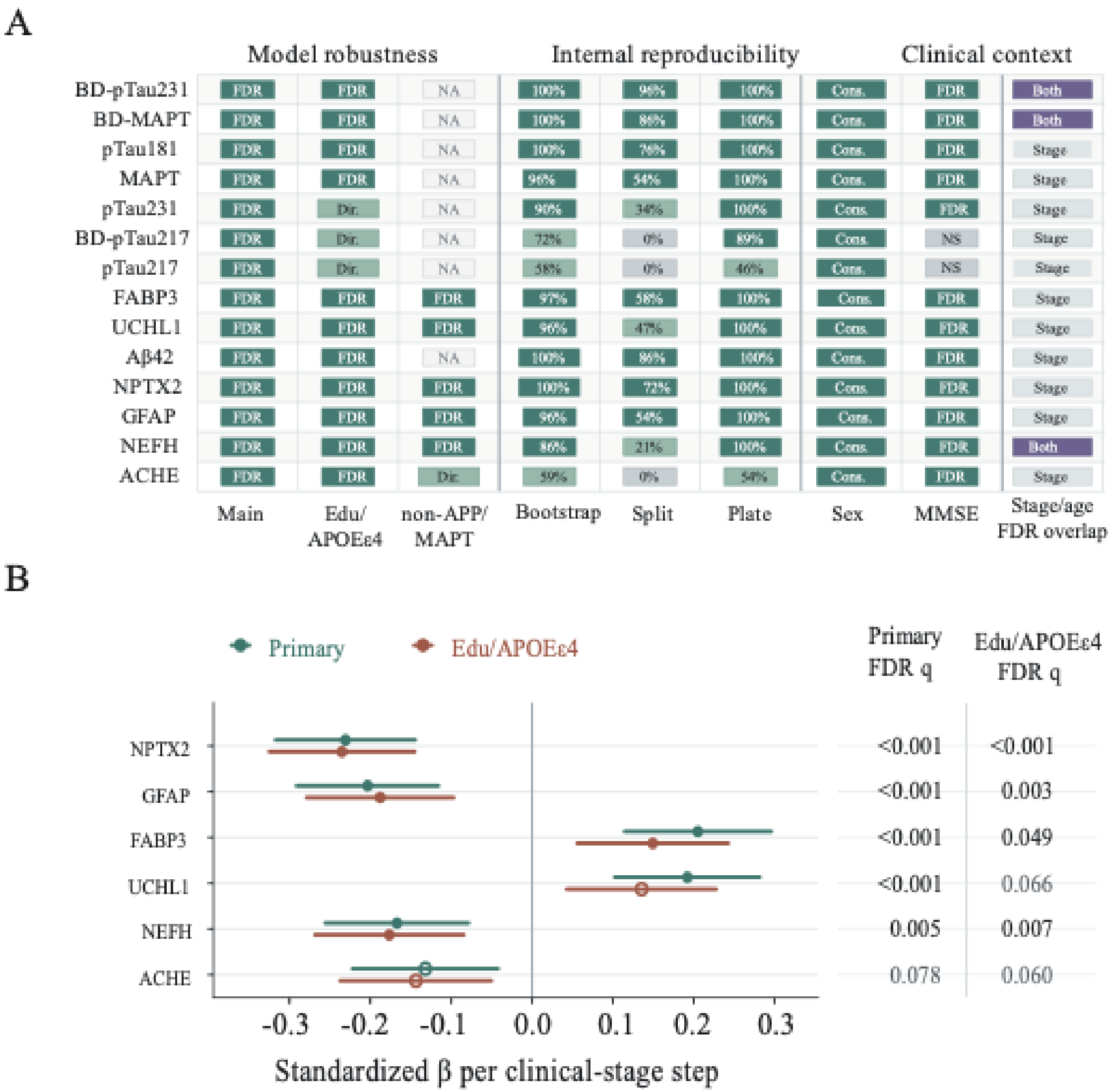

Supplementary Figure S1. (A) Evidence matrix across model robustness, internal reproducibility, and clinical-context analyses. FDR denotes $q < 0.05$; Dir., direction-consistent without FDR significance; Cons., concordant male/female directions without an FDR-significant interaction; NA, predefined APP/MAPT exclusion. Resampling cells show FDR-support frequency. (B) Non-APP/MAPT clinical-stage associations in primary and education/APOE ε4-adjusted models.

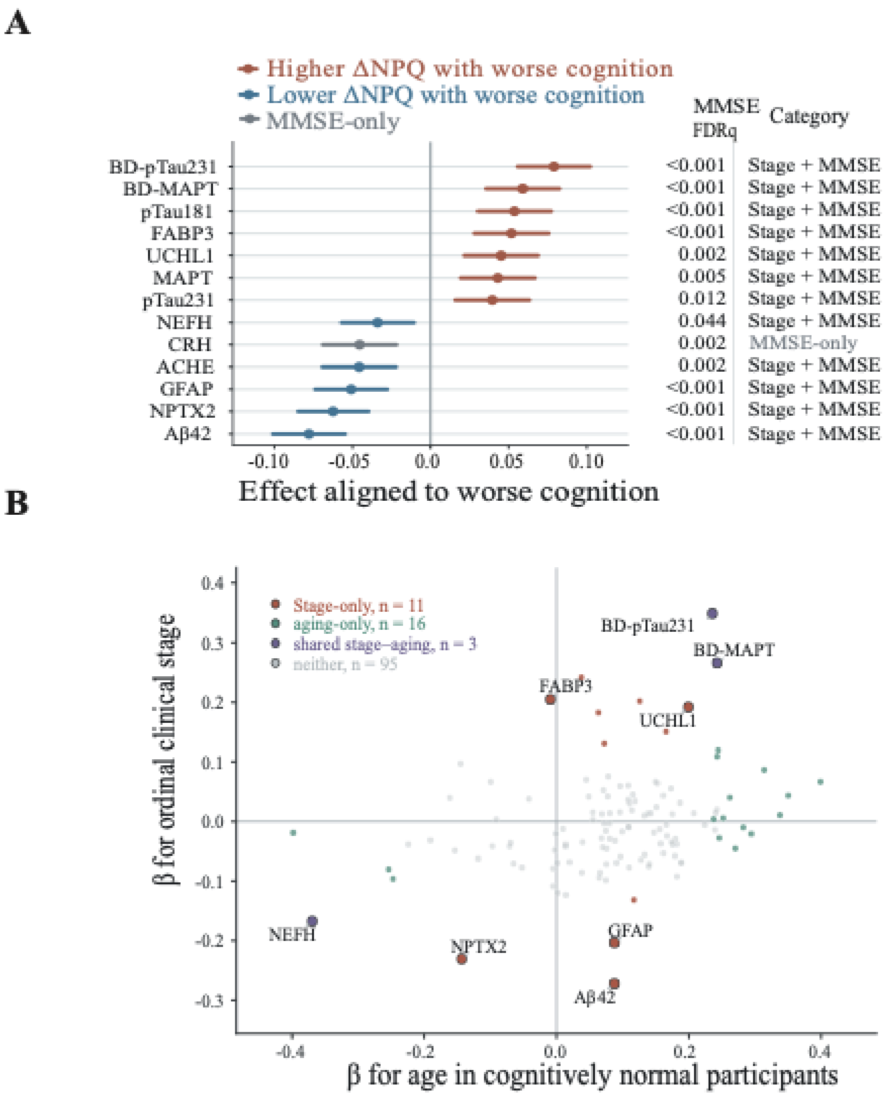

Supplementary Figure S2. (A) MMSE-associated effects aligned to worse cognition; the diagnosis-adjusted MMSE model yielded no FDR-significant readouts. (B) Cross-sectional ordinal clinical-stage effects versus age effects in cognitively normal participants. Filled and open points are defined in the panel keys; horizontal lines show 95% confidence intervals.

Supplementary Table S1

Population	N	Readouts	Same sign, n	Pearson q<0.05	Spearman q<0.05	Both q<0.05	Pearson only	Spearman only	Neither q<0.05	Median |rho − r|
**All subjects**	1199	125	118	65	62	59	6	3	57	0.012
**CN**	436	125	119	48	45	41	7	4	73	0.017
**MCI**	568	125	114	53	51	48	5	3	69	0.015
**AD**	195	125	114	25	25	21	4	4	96	0.022

Supplementary Table S2

analyte	Standardized β per one-category increase in clinical stage	SE	95% CI lower	95% CI upper	P value	FDR q value	Direction	FDR significant	Rank by FDR q	Rank by β
BD-pTau231	0.348720	0.044883	0.260659	0.436781	1.726E-14	2.158E-12	Positive association	Yes	1	125
Aβ42	-0.271996	0.044972	-0.360231	-0.183760	1.974E-09	1.234E-07	Negative association	Yes	2	1
BD-MAPT	0.266032	0.045659	0.176447	0.355616	7.328E-09	3.054E-07	Positive association	Yes	3	124
pTau181	0.241319	0.045561	0.151929	0.330709	1.410E-07	4.407E-06	Positive association	Yes	4	123
NPTX2	-0.230887	0.043977	-0.317172	-0.144603	1.806E-07	4.516E-06	Negative association	Yes	5	2
GFAP	-0.203364	0.044685	-0.291038	-0.115691	5.901E-06	1.229E-04	Negative association	Yes	6	3
FABP3	0.205245	0.046105	0.114788	0.295703	9.338E-06	1.668E-04	Positive association	Yes	7	122
MAPT	0.202055	0.045959	0.111882	0.292227	1.202E-05	1.878E-04	Positive association	Yes	8	121
UCHL1	0.192057	0.045681	0.102429	0.281684	2.821E-05	3.918E-04	Positive association	Yes	9	120
pTau231	0.182710	0.045945	0.092565	0.272855	7.421E-05	9.276E-04	Positive association	Yes	10	119
NEFH	-0.167083	0.045060	-0.255490	-0.078675	2.187E-04	2.486E-03	Negative association	Yes	11	4
BD-pTau217	0.150636	0.046077	0.060232	0.241041	1.110E-03	1.156E-02	Positive association	Yes	12	118
ACHE	-0.131957	0.045879	-0.221973	-0.041942	4.099E-03	3.941E-02	Negative association	Yes	13	5
pTau217	0.130342	0.046167	0.039761	0.220923	4.835E-03	4.317E-02	Positive association	Yes	14	117
CRH	-0.123573	0.045862	-0.213556	-0.033590	7.153E-03	5.961E-02	Negative association	No	15	6
DDC	0.119443	0.045288	0.030587	0.208299	8.466E-03	6.614E-02	Positive association	No	16	116
NPTX1	-0.120011	0.045929	-0.210124	-0.029899	9.091E-03	6.685E-02	Negative association	No	17	7
MDH1	0.108938	0.046082	0.018526	0.199351	1.824E-02	1.227E-01	Positive association	No	18	115
IL10	-0.108767	0.046170	-0.199354	-0.018181	1.865E-02	1.227E-01	Negative association	No	19	8
IL6R	-0.098718	0.046089	-0.189147	-0.008290	3.241E-02	2.002E-01	Negative association	No	20	9

FOLR1	-0.096351	0.045298	-0.185226	-0.007475	3.363E-02	2.002E-01	Negative association	No	21	10
CHIT1	0.096879	0.046252	0.006131	0.187627	3.643E-02	2.070E-01	Positive association	No	22	114
IGF1R	-0.094397	0.046178	-0.184998	-0.003796	4.116E-02	2.237E-01	Negative association	No	23	11
S100A12	-0.092383	0.046172	-0.182972	-0.001793	4.564E-02	2.377E-01	Negative association	No	24	12
SNCB	0.086391	0.045915	-0.003695	0.176477	6.015E-02	2.932E-01	Positive association	No	25	113
Aβ40	-0.085973	0.045840	-0.175911	0.003966	6.098E-02	2.932E-01	Negative association	No	26	13
IL12p70	-0.085818	0.046231	-0.176523	0.004887	6.366E-02	2.947E-01	Negative association	No	27	14
CCL11	-0.080287	0.045656	-0.169864	0.009289	7.892E-02	3.523E-01	Negative association	No	28	15
ApoE4	-0.078922	0.046245	-0.169656	0.011812	8.817E-02	3.798E-01	Negative association	No	29	16
IL6	-0.077267	0.046139	-0.167794	0.013259	9.427E-02	3.798E-01	Negative association	No	30	17
HBA1	-0.077239	0.046265	-0.168011	0.013533	9.529E-02	3.798E-01	Negative association	No	31	18
CCL13	-0.075520	0.045500	-0.164791	0.013751	9.723E-02	3.798E-01	Negative association	No	32	19
PDLIM5	0.075303	0.046166	-0.015276	0.165882	1.031E-01	3.907E-01	Positive association	No	33	112
PDGFRB	-0.071570	0.045531	-0.160903	0.017762	1.162E-01	4.105E-01	Negative association	No	34	20
VEGFA	-0.071320	0.045903	-0.161382	0.018742	1.205E-01	4.105E-01	Negative association	No	35	21
NGF	-0.070634	0.046005	-0.160895	0.019628	1.250E-01	4.105E-01	Negative association	No	36	22
BASP1	0.069906	0.046510	-0.021347	0.161158	1.331E-01	4.105E-01	Positive association	No	37	111
IL4	-0.069465	0.046354	-0.160412	0.021483	1.343E-01	4.105E-01	Negative association	No	38	23
PSEN1	-0.069192	0.046176	-0.159790	0.021406	1.343E-01	4.105E-01	Negative association	No	39	24
CCL22	-0.068991	0.045438	-0.158141	0.020159	1.292E-01	4.105E-01	Negative association	No	40	25
NPY	-0.068947	0.046055	-0.159308	0.021414	1.347E-01	4.105E-01	Negative association	No	41	26
IL2	-0.068203	0.046137	-0.158725	0.022318	1.396E-01	4.155E-01	Negative association	No	42	27

CCL3	0.066458	0.045978	-0.023750	0.156667	1.486E-01	4.217E-01	Positive association	No	43	110
VSNL1	0.066416	0.045957	-0.023751	0.156584	1.487E-01	4.217E-01	Positive association	No	44	109
SMOC1	0.066334	0.046429	-0.024761	0.157429	1.534E-01	4.217E-01	Positive association	No	45	108
VCAM1	-0.064499	0.045346	-0.153468	0.024470	1.552E-01	4.217E-01	Negative association	No	46	28
ENO2	0.063745	0.046195	-0.026891	0.154380	1.679E-01	4.465E-01	Positive association	No	47	107
YWHAZ	-0.062300	0.046104	-0.152756	0.028157	1.769E-01	4.606E-01	Negative association	No	48	29
TREM1	0.060109	0.046200	-0.030536	0.150754	1.935E-01	4.936E-01	Positive association	No	49	106
AGRN	-0.059105	0.046190	-0.149731	0.031521	2.009E-01	4.952E-01	Negative association	No	50	30
IL9	0.058977	0.046201	-0.031670	0.149623	2.020E-01	4.952E-01	Positive association	No	51	105
BACE1	-0.058025	0.045947	-0.148173	0.032122	2.069E-01	4.973E-01	Negative association	No	52	31
Aβ38	-0.055864	0.045901	-0.145922	0.034194	2.238E-01	5.279E-01	Negative association	No	53	32
NRGN	0.054996	0.046510	-0.036257	0.146249	2.373E-01	5.472E-01	Positive association	No	54	104
TEK	-0.053734	0.045785	-0.143565	0.036096	2.408E-01	5.472E-01	Negative association	No	55	33
SFRP1	0.052252	0.046189	-0.038372	0.142876	2.582E-01	5.763E-01	Positive association	No	56	103
IL33	-0.048810	0.045903	-0.138871	0.041252	2.879E-01	6.313E-01	Negative association	No	57	34
SFTPD	-0.048143	0.046366	-0.139113	0.042828	2.993E-01	6.451E-01	Negative association	No	58	35
POSTN	-0.045463	0.045315	-0.134371	0.043445	3.159E-01	6.694E-01	Negative association	No	59	37
CX3CL1	-0.045762	0.046318	-0.136639	0.045115	3.234E-01	6.708E-01	Negative association	No	60	36
GDF15	0.044681	0.046165	-0.045895	0.135257	3.333E-01	6.708E-01	Positive association	No	61	102
CXCL8	-0.043724	0.046147	-0.134265	0.046817	3.436E-01	6.708E-01	Negative association	No	62	38
IL18	0.043088	0.045529	-0.046240	0.132416	3.441E-01	6.708E-01	Positive association	No	63	101
SNCA	0.043021	0.046287	-0.047795	0.133836	3.529E-01	6.708E-01	Positive association	No	64	100

TNF	-0.041938	0.046084	-0.132355	0.048479	3.630E-01	6.708E-01	Negative association	No	65	39
VEGFD	-0.041833	0.045414	-0.130935	0.047270	3.572E-01	6.708E-01	Negative association	No	66	40
IL17A	-0.041659	0.046401	-0.132698	0.049381	3.695E-01	6.708E-01	Negative association	No	67	41
FGF2	0.041650	0.046384	-0.049355	0.132655	3.694E-01	6.708E-01	Positive association	No	68	99
CNTN2	0.041086	0.045839	-0.048850	0.131022	3.703E-01	6.708E-01	Positive association	No	69	98
GOT1	0.039717	0.046252	-0.051031	0.130465	3.907E-01	6.779E-01	Positive association	No	70	97
NEFL	0.039336	0.045319	-0.049579	0.128252	3.856E-01	6.779E-01	Positive association	No	71	96
VGF	-0.038863	0.046441	-0.129981	0.052256	4.029E-01	6.779E-01	Negative association	No	72	42
MSLN	-0.038580	0.045208	-0.127278	0.050119	3.936E-01	6.779E-01	Negative association	No	73	43
REST	-0.038462	0.046165	-0.129038	0.052115	4.049E-01	6.779E-01	Negative association	No	74	44
CCL26	-0.038178	0.046106	-0.128639	0.052283	4.078E-01	6.779E-01	Negative association	No	75	45
TAFA5	-0.037515	0.046265	-0.128288	0.053258	4.176E-01	6.779E-01	Negative association	No	76	46
IL5	0.037389	0.045689	-0.052253	0.127031	4.133E-01	6.779E-01	Positive association	No	77	95
TREM2	-0.035977	0.045450	-0.125150	0.053196	4.288E-01	6.871E-01	Negative association	No	78	47
CD40LG	0.036130	0.046248	-0.054610	0.126870	4.348E-01	6.880E-01	Positive association	No	79	94
CHI3L1	-0.034852	0.045988	-0.125081	0.055376	4.487E-01	6.924E-01	Negative association	No	80	48
TIMP3	-0.034685	0.045568	-0.124090	0.054721	4.467E-01	6.924E-01	Negative association	No	81	49
YWHAG	0.034057	0.046395	-0.056971	0.125085	4.631E-01	7.059E-01	Positive association	No	82	93
CCL4	-0.032261	0.046352	-0.123204	0.058682	4.866E-01	7.241E-01	Negative association	No	83	50
IGFBP7	-0.031672	0.045367	-0.120682	0.057338	4.852E-01	7.241E-01	Negative association	No	84	51
KDR	0.031130	0.045942	-0.059008	0.121268	4.982E-01	7.326E-01	Positive association	No	85	92
GDI1	0.030151	0.046183	-0.060461	0.120762	5.140E-01	7.471E-01	Positive association	No	86	91

SQSTM1	-0.029364	0.046269	-0.120144	0.061416	5.258E-01	7.519E-01	Negative association	No	87	52
SLIT2	-0.029123	0.046282	-0.119929	0.061684	5.293E-01	7.519E-01	Negative association	No	88	53
ARSA	-0.028212	0.046285	-0.119023	0.062600	5.423E-01	7.532E-01	Negative association	No	89	54
CCL2	-0.027874	0.045691	-0.117519	0.061772	5.419E-01	7.532E-01	Negative association	No	90	55
TARDBP	-0.024405	0.046426	-0.115493	0.066683	5.992E-01	8.177E-01	Negative association	No	91	56
PRDX6	0.024150	0.046271	-0.066634	0.114933	6.018E-01	8.177E-01	Positive association	No	92	90
IL16	-0.020915	0.045866	-0.110905	0.069076	6.485E-01	8.639E-01	Negative association	No	93	57
CALB2	-0.020755	0.045675	-0.110370	0.068860	6.496E-01	8.639E-01	Negative association	No	94	58
IL7	0.020217	0.045646	-0.069340	0.109775	6.579E-01	8.657E-01	Positive association	No	95	89
CST3	-0.019009	0.045150	-0.107594	0.069576	6.738E-01	8.774E-01	Negative association	No	96	59
IFNG	0.019006	0.046248	-0.071734	0.109745	6.812E-01	8.776E-01	Positive association	No	97	88
IL1B	-0.018581	0.046265	-0.109353	0.072191	6.880E-01	8.776E-01	Negative association	No	98	60
FLT1	-0.016989	0.045770	-0.106791	0.072813	7.106E-01	8.805E-01	Negative association	No	99	61
BD-pTau181	-0.015802	0.046357	-0.106754	0.075150	7.333E-01	8.805E-01	Negative association	No	100	62
UBB	0.015721	0.046133	-0.074793	0.106235	7.333E-01	8.805E-01	Positive association	No	101	87
PTN	0.015465	0.045487	-0.073781	0.104710	7.339E-01	8.805E-01	Positive association	No	102	86
CXCL1	0.014651	0.045890	-0.075386	0.104688	7.496E-01	8.805E-01	Positive association	No	103	85
CCL17	-0.014367	0.046154	-0.104921	0.076188	7.556E-01	8.805E-01	Negative association	No	104	63
PGK1	0.013943	0.046376	-0.077048	0.104934	7.637E-01	8.805E-01	Positive association	No	105	84
CXCL10	0.013909	0.045979	-0.076303	0.104121	7.623E-01	8.805E-01	Positive association	No	106	83
S100B	0.013789	0.045903	-0.076272	0.103851	7.639E-01	8.805E-01	Positive association	No	107	82
CSF2	0.013339	0.045931	-0.076777	0.103456	7.715E-01	8.805E-01	Positive association	No	108	81

PGF	-0.013315	0.044481	-0.100587	0.073958	7.647E-01	8.805E-01	Negative association	No	109	64
pTDP43-409	0.012723	0.046258	-0.078036	0.103482	7.833E-01	8.805E-01	Positive association	No	110	80
SOD1	0.012528	0.046081	-0.077883	0.102938	7.858E-01	8.805E-01	Positive association	No	111	79
Oligo-SNCA	-0.012438	0.046458	-0.103589	0.078714	7.890E-01	8.805E-01	Negative association	No	112	65
IL15	0.010768	0.045587	-0.078675	0.100210	8.133E-01	8.965E-01	Positive association	No	113	78
CD63	-0.010031	0.045964	-0.100212	0.080150	8.273E-01	8.965E-01	Negative association	No	114	66
KLK6	0.009847	0.046147	-0.080694	0.100388	8.311E-01	8.965E-01	Positive association	No	115	77
RUVBL2	-0.009817	0.046251	-0.100562	0.080928	8.320E-01	8.965E-01	Negative association	No	116	67
NPTXR	-0.009192	0.046263	-0.099961	0.081577	8.425E-01	9.001E-01	Negative association	No	117	68
ICAM1	0.005751	0.045058	-0.082653	0.094155	8.985E-01	9.455E-01	Positive association	No	118	76
ANXA5	-0.005574	0.046277	-0.096369	0.085221	9.041E-01	9.455E-01	Negative association	No	119	69
HTT	-0.005298	0.046216	-0.095976	0.085379	9.087E-01	9.455E-01	Negative association	No	120	70
SNAP25	0.003936	0.045852	-0.086027	0.093899	9.316E-01	9.455E-01	Positive association	No	121	75
pSNCA-129	-0.003467	0.046481	-0.094664	0.087729	9.405E-01	9.455E-01	Negative association	No	122	71
IL13	0.003384	0.046224	-0.087307	0.094076	9.416E-01	9.455E-01	Positive association	No	123	74
GDNF	-0.003355	0.046107	-0.093817	0.087108	9.420E-01	9.455E-01	Negative association	No	124	72
PARK7	0.003154	0.046165	-0.087422	0.093730	9.455E-01	9.455E-01	Positive association	No	125	73

Supplementary Table S3

analyte	CSF standardized β	CSF P	CSF FDR q	Plasma standardized β	Plasma P	Plasma FDR q	ΔNPQ standardized β	ΔNPQ P	ΔNPQ FDR q
Aβ42	-0.318008	3.752E-14	4.263E-13	-0.070108	1.243E-01	2.956E-01	-0.271996	1.974E-09	1.234E-07
UCHL1	0.296542	8.324E-13	8.670E-12	0.087769	5.476E-02	1.801E-01	0.192057	2.821E-05	3.918E-04
FABP3	0.229158	2.331E-08	1.942E-07	0.020852	6.246E-01	8.131E-01	0.205245	9.338E-06	1.668E-04
GFAP	0.097907	1.582E-02	5.994E-02	0.402756	4.916E-21	6.827E-20	-0.203364	5.901E-06	1.229E-04
BD-pTau217	0.531983	3.325E-39	4.156E-37	0.658216	9.438E-54	1.180E-51	0.150636	1.110E-03	1.156E-02
BD-pTau231	0.498854	2.109E-34	6.590E-33	0.606997	4.508E-45	1.879E-43	0.348720	1.726E-14	2.158E-12
pTau217	0.495133	6.200E-34	1.550E-32	0.589074	4.997E-44	1.561E-42	0.130342	4.835E-03	4.317E-02
ACHE	0.477937	5.168E-31	9.228E-30	0.592183	1.713E-42	4.283E-41	-0.131957	4.099E-03	3.941E-02
pTau231	0.477489	1.660E-31	3.459E-30	0.522734	1.503E-35	3.131E-34	0.182710	7.421E-05	9.276E-04
BD-MAPT	0.421977	1.902E-24	2.972E-23	0.538600	1.625E-34	2.901E-33	0.266032	7.328E-09	3.054E-07
pTau181	0.501806	1.068E-34	4.452E-33	0.458521	2.588E-27	4.044E-26	0.241319	1.410E-07	4.407E-06
MAPT	0.409625	3.561E-23	4.946E-22	0.364866	8.003E-17	9.095E-16	0.202055	1.202E-05	1.878E-04
NPTX2	-0.183785	1.385E-05	1.082E-04	0.196841	2.698E-06	2.249E-05	-0.230887	1.806E-07	4.516E-06
NEFH	0.109834	5.400E-03	2.250E-02	0.206988	2.287E-06	2.042E-05	-0.167083	2.187E-04	2.486E-03
BD-pTau181	0.511253	5.805E-36	3.628E-34	0.634014	5.448E-50	3.405E-48	-0.015802	7.333E-01	8.805E-01
ApoE4	0.351655	3.148E-17	3.935E-16	0.397953	4.277E-19	5.347E-18	-0.078922	8.817E-02	3.798E-01
NEFL	0.265827	1.282E-11	1.233E-10	0.321172	2.170E-15	2.261E-14	0.039336	3.856E-01	6.779E-01
SMOC1	0.240691	1.084E-08	9.679E-08	0.125104	5.985E-03	2.878E-02	0.066334	1.534E-01	4.217E-01
NPTXR	-0.163454	1.187E-04	7.416E-04	-0.143896	1.519E-03	8.628E-03	-0.009192	8.425E-01	9.001E-01
TREM1	0.148939	2.083E-04	1.183E-03	0.148566	3.906E-04	2.872E-03	0.060109	1.935E-01	4.936E-01
VSNL1	0.149295	4.117E-04	2.238E-03	0.147438	1.145E-03	7.158E-03	0.066416	1.487E-01	4.217E-01
CHI3L1	0.125980	1.795E-03	8.630E-03	0.119023	6.540E-03	3.028E-02	-0.034852	4.487E-01	6.924E-01
NRGN	0.176571	2.938E-05	2.160E-04	0.007136	8.766E-01	9.208E-01	0.054996	2.373E-01	5.472E-01
SNCB	0.171777	4.416E-05	3.066E-04	0.053610	2.431E-01	4.469E-01	0.086391	6.015E-02	2.932E-01

PDLIM5	0.164639	9.328E-05	6.137E-04	-0.035873	4.349E-01	6.795E-01	0.075303	1.031E-01	3.907E-01
MDH1	0.160455	1.474E-04	8.776E-04	0.010446	8.201E-01	8.914E-01	0.108938	1.824E-02	1.227E-01
CHIT1	0.140930	8.077E-04	4.038E-03	0.049165	2.779E-01	4.892E-01	0.096879	3.643E-02	2.070E-01
DDC	0.140584	7.770E-04	4.038E-03	0.005209	9.091E-01	9.314E-01	0.119443	8.466E-03	6.614E-02
GDI1	0.124403	3.031E-03	1.403E-02	0.050712	2.700E-01	4.821E-01	0.030151	5.140E-01	7.471E-01
ENO2	0.122105	3.831E-03	1.710E-02	0.019308	6.745E-01	8.131E-01	0.063745	1.679E-01	4.465E-01
PGK1	0.119894	4.391E-03	1.893E-02	0.030907	5.011E-01	7.407E-01	0.013943	7.637E-01	8.805E-01
IGF1R	0.016818	6.910E-01	7.924E-01	0.255262	6.907E-09	6.642E-08	-0.094397	4.116E-02	2.237E-01
VCAM1	0.008017	8.427E-01	9.081E-01	0.205532	2.926E-06	2.286E-05	-0.064499	1.552E-01	4.217E-01
SLIT2	0.091151	3.112E-02	1.024E-01	0.159957	4.383E-04	3.044E-03	-0.029123	5.293E-01	7.519E-01
BACE1	-0.012968	7.606E-01	8.565E-01	0.147163	9.189E-04	6.046E-03	-0.058025	2.069E-01	4.973E-01
SNAP25	0.058126	1.672E-01	3.484E-01	0.145123	1.488E-03	8.628E-03	0.003936	9.316E-01	9.455E-01
CCL11	0.034877	4.076E-01	6.379E-01	0.129977	3.157E-03	1.716E-02	-0.080287	7.892E-02	3.523E-01
FLT1	0.061320	1.472E-01	3.173E-01	0.132736	3.688E-03	1.921E-02	-0.016989	7.106E-01	8.805E-01
S100A12	0.017481	6.812E-01	7.884E-01	0.130375	4.277E-03	2.139E-02	-0.092383	4.564E-02	2.377E-01
NPTX1	-0.080191	5.923E-02	1.645E-01	0.114750	1.071E-02	4.783E-02	-0.120011	9.091E-03	6.685E-02
IL7	0.102715	1.265E-02	5.099E-02	0.040990	3.708E-01	6.099E-01	0.020217	6.579E-01	8.657E-01
VEGFD	-0.103142	1.498E-02	5.850E-02	0.021198	6.371E-01	8.131E-01	-0.041833	3.572E-01	6.708E-01
CRH	-0.064709	1.288E-01	2.938E-01	0.079119	8.306E-02	2.227E-01	-0.123573	7.153E-03	5.961E-02
PDGFRB	-0.011062	7.941E-01	8.784E-01	0.110121	1.654E-02	7.128E-02	-0.071570	1.162E-01	4.105E-01
GOT1	0.097710	2.124E-02	7.808E-02	0.081382	7.493E-02	2.178E-01	0.039717	3.907E-01	6.779E-01
ANXA5	0.092915	2.308E-02	8.242E-02	0.048443	2.922E-01	5.003E-01	-0.005574	9.041E-01	9.455E-01
IGFBP7	0.036090	3.926E-01	6.373E-01	0.099794	2.133E-02	8.334E-02	-0.031672	4.852E-01	7.241E-01
IL33	0.035671	4.016E-01	6.379E-01	0.103308	2.078E-02	8.334E-02	-0.048810	2.879E-01	6.313E-01

POSTN	-0.000099	9.980E-01	9.990E-01	0.103258	2.092E-02	8.334E-02	-0.045463	3.159E-01	6.694E-01
IL6R	-0.095513	2.499E-02	8.675E-02	0.070814	1.231E-01	2.956E-01	-0.098718	3.241E-02	2.002E-01
CCL13	-0.044071	2.895E-01	5.096E-01	0.103067	2.431E-02	9.209E-02	-0.075520	9.723E-02	3.798E-01
IL12p70	-0.092474	2.849E-02	9.624E-02	0.020782	6.486E-01	8.131E-01	-0.085818	6.366E-02	2.947E-01
CCL2	0.032954	4.167E-01	6.423E-01	0.100981	2.749E-02	9.861E-02	-0.027874	5.419E-01	7.532E-01
FOLR1	-0.063635	1.316E-01	2.938E-01	0.096355	2.761E-02	9.861E-02	-0.096351	3.363E-02	2.002E-01
pSNCA-129	0.090537	3.315E-02	1.036E-01	0.028158	5.402E-01	7.673E-01	-0.003467	9.405E-01	9.455E-01
SNCA	0.090467	3.272E-02	1.036E-01	0.016836	7.143E-01	8.428E-01	0.043021	3.529E-01	6.708E-01
YWHAG	0.088047	3.751E-02	1.144E-01	0.036677	4.248E-01	6.774E-01	0.034057	4.631E-01	7.059E-01
IL10	-0.078322	6.309E-02	1.714E-01	0.056066	2.197E-01	4.224E-01	-0.108767	1.865E-02	1.227E-01
NGF	0.036860	3.798E-01	6.247E-01	0.095445	3.550E-02	1.231E-01	-0.070634	1.250E-01	4.105E-01
TREM2	0.054077	1.921E-01	3.700E-01	0.091205	3.644E-02	1.231E-01	-0.035977	4.288E-01	6.871E-01
NPY	-0.085061	4.456E-02	1.326E-01	0.030628	5.004E-01	7.407E-01	-0.068947	1.347E-01	4.105E-01
pTDP43-409	0.082038	5.297E-02	1.540E-01	0.015339	7.370E-01	8.453E-01	0.012723	7.833E-01	8.805E-01
SFRP1	0.081376	5.577E-02	1.584E-01	0.020288	6.577E-01	8.131E-01	0.052252	2.582E-01	5.763E-01
Aβ40	-0.076488	7.178E-02	1.909E-01	0.080617	7.491E-02	2.178E-01	-0.085973	6.098E-02	2.932E-01
IL4	-0.022187	6.022E-01	7.528E-01	0.085822	6.164E-02	1.976E-01	-0.069465	1.343E-01	4.105E-01
PSEN1	-0.074687	7.685E-02	2.001E-01	0.067945	1.370E-01	3.054E-01	-0.069192	1.343E-01	4.105E-01
IL6	-0.012102	7.718E-01	8.614E-01	0.084021	6.530E-02	2.041E-01	-0.077267	9.427E-02	3.798E-01
Aβ38	-0.072961	8.565E-02	2.141E-01	0.020589	6.513E-01	8.131E-01	-0.055864	2.238E-01	5.279E-01
TNF	-0.072716	8.398E-02	2.141E-01	0.018779	6.765E-01	8.131E-01	-0.041938	3.630E-01	6.708E-01
REST	0.022897	5.903E-01	7.528E-01	0.081440	7.223E-02	2.178E-01	-0.038462	4.049E-01	6.779E-01
CX3CL1	-0.018633	6.614E-01	7.884E-01	0.080273	7.676E-02	2.181E-01	-0.045762	3.234E-01	6.708E-01

CCL4	-0.072153	8.905E-02	2.183E-01	-0.015352	7.381E-01	8.453E-01	-0.032261	4.866E-01	7.241E-01
IL9	-0.021129	6.132E-01	7.589E-01	-0.078457	7.951E-02	2.208E-01	0.058977	2.020E-01	4.952E-01
VEGFA	-0.063605	1.308E-01	2.938E-01	0.078739	8.374E-02	2.227E-01	-0.071320	1.205E-01	4.105E-01
FGF2	0.069671	1.009E-01	2.426E-01	-0.019816	6.665E-01	8.131E-01	0.041650	3.694E-01	6.708E-01
AGRN	-0.053595	2.032E-01	3.792E-01	0.074918	9.339E-02	2.432E-01	-0.059105	2.009E-01	4.952E-01
IL15	0.033540	4.083E-01	6.379E-01	0.074204	1.020E-01	2.602E-01	0.010768	8.133E-01	8.965E-01
MSLN	-0.067944	1.104E-01	2.604E-01	-0.008631	8.467E-01	9.046E-01	-0.038580	3.936E-01	6.779E-01
SQSTM1	0.017166	6.798E-01	7.884E-01	0.068354	1.293E-01	2.956E-01	-0.029364	5.258E-01	7.519E-01
TAFA5	-0.030374	4.701E-01	6.754E-01	0.068841	1.279E-01	2.956E-01	-0.037515	4.176E-01	6.779E-01
TEK	-0.044754	2.891E-01	5.096E-01	0.070287	1.234E-01	2.956E-01	-0.053734	2.408E-01	5.472E-01
YWHAZ	0.001959	9.632E-01	9.833E-01	0.069188	1.301E-01	2.956E-01	-0.062300	1.769E-01	4.606E-01
BASP1	0.026523	5.324E-01	7.397E-01	-0.067996	1.393E-01	3.054E-01	0.069906	1.331E-01	4.105E-01
CCL22	-0.024990	5.530E-01	7.485E-01	0.065859	1.445E-01	3.114E-01	-0.068991	1.292E-01	4.105E-01
IL18	0.059810	1.465E-01	3.173E-01	0.036284	4.232E-01	6.774E-01	0.043088	3.441E-01	6.708E-01
CST3	0.060350	1.552E-01	3.287E-01	0.061395	1.631E-01	3.398E-01	-0.019009	6.738E-01	8.774E-01
HBA1	-0.024094	5.707E-01	7.528E-01	0.063539	1.627E-01	3.398E-01	-0.077239	9.529E-02	3.798E-01
GDF15	0.056428	1.705E-01	3.494E-01	-0.002499	9.565E-01	9.642E-01	0.044681	3.333E-01	6.708E-01
CNTN2	-0.057253	1.765E-01	3.507E-01	-0.058047	2.023E-01	4.014E-01	0.041086	3.703E-01	6.708E-01
UBB	0.057483	1.768E-01	3.507E-01	-0.015022	7.439E-01	8.453E-01	0.015721	7.333E-01	8.805E-01
ICAM1	0.001841	9.645E-01	9.833E-01	0.061329	1.798E-01	3.684E-01	0.005751	8.985E-01	9.455E-01
IL2	-0.055189	1.924E-01	3.700E-01	0.049084	2.838E-01	4.927E-01	-0.068203	1.396E-01	4.155E-01
TIMP3	0.041773	3.194E-01	5.469E-01	0.060541	1.841E-01	3.711E-01	-0.034685	4.467E-01	6.924E-01
KLK6	-0.054062	2.024E-01	3.792E-01	-0.013929	7.610E-01	8.553E-01	0.009847	8.311E-01	8.965E-01
VGF	0.052683	2.146E-01	3.945E-01	0.042470	3.557E-01	5.928E-01	-0.038863	4.029E-01	6.779E-01

CALB2	-0.005433	8.971E-01	9.556E-01	0.056086	2.116E-01	4.133E-01	-0.020755	6.496E-01	8.639E-01
CCL3	0.017445	6.676E-01	7.884E-01	-0.028012	5.364E-01	7.673E-01	0.066458	1.486E-01	4.217E-01
IFNG	-0.026461	5.326E-01	7.397E-01	-0.055182	2.288E-01	4.334E-01	0.019006	6.812E-01	8.776E-01
IL17A	0.001726	9.676E-01	9.833E-01	0.053695	2.422E-01	4.469E-01	-0.041659	3.695E-01	6.708E-01
IL16	0.000053	9.990E-01	9.990E-01	0.051407	2.598E-01	4.706E-01	-0.020915	6.485E-01	8.639E-01
RUVBL2	0.046679	2.724E-01	4.934E-01	0.030265	5.096E-01	7.407E-01	-0.009817	8.320E-01	8.965E-01
CXCL8	-0.032300	4.405E-01	6.555E-01	0.047463	3.007E-01	5.079E-01	-0.043724	3.436E-01	6.708E-01
GDNF	0.042999	3.120E-01	5.416E-01	0.008157	8.584E-01	9.093E-01	-0.003355	9.420E-01	9.455E-01
CD40LG	0.038463	3.586E-01	6.057E-01	-0.005790	8.997E-01	9.295E-01	0.036130	4.348E-01	6.880E-01
KDR	-0.037521	3.744E-01	6.240E-01	-0.036279	4.281E-01	6.774E-01	0.031130	4.982E-01	7.326E-01
CD63	-0.033144	4.214E-01	6.423E-01	0.020307	6.575E-01	8.131E-01	-0.010031	8.273E-01	8.965E-01
SFTPD	-0.033660	4.291E-01	6.463E-01	0.031995	4.792E-01	7.395E-01	-0.048143	2.993E-01	6.451E-01
CSF2	-0.030411	4.679E-01	6.754E-01	-0.021416	6.367E-01	8.131E-01	0.013339	7.715E-01	8.805E-01
SOD1	0.030858	4.660E-01	6.754E-01	0.026187	5.677E-01	7.973E-01	0.012528	7.858E-01	8.805E-01
CCL26	-0.008406	8.427E-01	9.081E-01	0.019959	6.596E-01	8.131E-01	-0.038178	4.078E-01	6.779E-01
IL5	0.022390	5.973E-01	7.528E-01	-0.016304	7.147E-01	8.428E-01	0.037389	4.133E-01	6.779E-01
HTT	0.029332	4.833E-01	6.865E-01	0.011369	8.044E-01	8.890E-01	-0.005298	9.087E-01	9.455E-01
CCL17	-0.021646	5.945E-01	7.528E-01	0.031782	4.892E-01	7.407E-01	-0.014367	7.556E-01	8.805E-01
TARDBP	-0.002843	9.467E-01	9.833E-01	0.030364	5.084E-01	7.407E-01	-0.024405	5.992E-01	8.177E-01
CXCL1	0.025472	5.408E-01	7.428E-01	0.006324	8.900E-01	9.271E-01	0.014651	7.496E-01	8.805E-01
Oligo-SNCA	0.024962	5.569E-01	7.485E-01	0.020862	6.501E-01	8.131E-01	-0.012438	7.890E-01	8.805E-01
IL13	0.022463	5.971E-01	7.528E-01	0.013551	7.664E-01	8.553E-01	0.003384	9.416E-01	9.455E-01
S100B	0.023002	5.837E-01	7.528E-01	0.009449	8.369E-01	9.018E-01	0.013789	7.639E-01	8.805E-01
ARSA	-0.004819	9.098E-01	9.556E-01	0.022449	6.247E-01	8.131E-01	-0.028212	5.423E-01	7.532E-01

PRDX6	0.020893	6.213E-01	7.614E-01	-0.024008	6.003E-01	8.131E-01	0.024150	6.018E-01	8.177E-01
PGF	-0.018488	6.390E-01	7.755E-01	0.003992	9.300E-01	9.451E-01	-0.013315	7.647E-01	8.805E-01
PARK7	-0.017826	6.747E-01	7.884E-01	0.001771	9.692E-01	9.692E-01	0.003154	9.455E-01	9.455E-01
IL1B	0.016253	7.025E-01	7.983E-01	0.025545	5.783E-01	8.033E-01	-0.018581	6.880E-01	8.776E-01
PTN	0.004970	9.070E-01	9.556E-01	0.015733	7.266E-01	8.453E-01	0.015465	7.339E-01	8.805E-01
CXCL10	-0.009721	8.171E-01	8.959E-01	-0.010801	8.108E-01	8.890E-01	0.013909	7.623E-01	8.805E-01

Supplementary Table S4

Variable	Overall MCI survival cohort	Non-converters	Converters	P value
Participants, n	547	344	203	
Age, years	72.4 (7.6)	71.6 (7.7)	73.6 (7.1)	0.002
Female sex, n (%)	221 (40.4%)	143 (41.6%)	78 (38.4%)	0.528
Education, years	16.1 (2.7)	16.2 (2.7)	15.9 (2.8)	0.140
APOE ε4 carrier, n (%)	274 (50.1%)	139 (40.4%)	135 (66.5%)	<0.001
Baseline MMSE	27.7 (1.8)	28.1 (1.7)	27.0 (1.9)	<0.001
Baseline CDR-SB	1.5 [1.0, 2.0]	1.0 [0.5, 1.5]	2.0 [1.0, 2.5]	<0.001
CSF Aβ42/40-derived amyloid burden, z score	0.11 (0.99)	-0.08 (0.97)	0.44 (0.94)	<0.001
CSF pTau217, NPQ	15.59 (1.44)	15.18 (1.37)	16.29 (1.28)	<0.001
Follow-up duration, years	3.0 [1.6, 5.3]	4.0 [2.2, 6.4]	2.0 [1.0, 3.1]	<0.001
AD dementia conversion events, n (%)	203 (37.1%)	0 (0.0%)	203 (100.0%)	
Non-AD dementia competing events, n (%)	9 (1.6%)	9 (2.6%)	0 (0.0%)	
Censored without AD dementia, n (%)	335 (61.2%)	335 (97.4%)	0 (0.0%)	

Supplementary Table S5

Covariate set	Comparison	Corrected ΔC-index	Paired optimism-corrected bootstrap 95% CI
**Clinical**	ΔNPQ – CSF-only	-0.0040	−0.0156 to 0.0062
	ΔNPQ – Plasma-only	0.0044	−0.0171 to 0.0233
	ΔNPQ – Joint	-0.0140	−0.0278 to 0.0008
	Plasma-only – CSF-only	-0.0084	−0.0250 to 0.0095
	Plasma-only – Joint	-0.0183	−0.0300 to −0.0032
	CSF-only – Joint	-0.0100	−0.0165 to 0.0003
**Amyloid/tau-adjusted**	ΔNPQ – CSF-only	-0.0014	−0.0094 to 0.0049
	ΔNPQ – Plasma-only	-0.0058	−0.0172 to 0.0058
	ΔNPQ – Joint	-0.0094	−0.0200 to 0.0031
	Plasma-only – CSF-only	0.0045	−0.0065 to 0.0128
	Plasma-only – Joint	-0.0035	−0.0074 to 0.0033
	CSF-only – Joint	-0.0080	−0.0141 to 0.0027

Supplementary Table S6

Analysis cohort	Readout / score	Gene target	N	Base R²	Full R²	ΔR²	Partial R²	Nested F-test P
Full paired cohort	BD-pTau231	MAPT	1199	0.034	0.862	0.828	0.857	<0.001
	BD-MAPT	MAPT	1199	0.022	0.682	0.661	0.675	<0.001
	BD-pTau217	MAPT	1199	0.023	0.613	0.590	0.604	<0.001
	MAPT	MAPT	1199	0.021	0.577	0.556	0.568	<0.001
	pTau181	MAPT	1199	0.031	0.656	0.625	0.645	<0.001
	pTau217	MAPT	1199	0.022	0.598	0.576	0.589	<0.001
	pTau231	MAPT	1199	0.025	0.652	0.628	0.643	<0.001
	Aβ42	APP	1199	0.049	0.196	0.147	0.155	<0.001
	NPTX2	NPTX2	1199	0.097	0.123	0.026	0.029	<0.001
	GFAP	GFAP	1199	0.073	0.093	0.020	0.021	<0.001
	FABP3	FABP3	1199	0.014	0.309	0.295	0.299	<0.001
	UCHL1	UCHL1	1199	0.034	0.175	0.141	0.146	<0.001
	NEFH	NEFH	1199	0.063	0.064	0.001	0.001	0.721
	ACHE	ACHE	1199	0.034	0.098	0.064	0.066	<0.001
	Six-readout ΔNPQ score	Composit e	1199	0.0673 74781	0.5258 10731	0.4584 3595	0.4915 54314	<0.001
**MCI follow-up cohort**	Six-readout ΔNPQ score	Composit e	547	0.1173 24028	0.5499 34772	0.4326 10744	0.4901 12746	<0.001

Supplementary Table S7

Covariate set	Added score	HR per 1 SD (95% CI)	Cox P	Corrected C-index	Corrected ΔC-index	ΔAIC	LRT P
**Clinical**	Base model	—	—	0.7641112 68		0	
**Clinical**	Original 6-analyte ΔNPQ	1.64 (1.38–1.96)	2.20 × 10^{-8}	0.7848622 23	0.0207509 56	- 29.79 8368	1.71 × 10^{-8}
**Clinical**	CN/AD-derived 10-analyte ΔNPQ	1.46 (1.22–1.75)	3.94 × 10^{-5}	0.7763419 81	0.0122307 13	- 15.02 72808	3.68 × 10^{-5}
**Amyloid/tau-adjusted**	Base model	—	—	0.7843978 87		0	

**Amyloid/tau-adjusted**	Original 6-analyte ΔNPQ	1.26 (1.01–1.58)	0.044	0.786645004	0.002247118	-2.09477712	0.043
**Amyloid/tau-adjusted**	CN/AD-derived 10-analyte ΔNPQ	1.17 (0.96–1.43)	0.114	0.785007274	0.000609387	-0.50118634	0.114

References

1. The GTEx Consortium atlas of genetic regulatory effects across human tissues. *Science* 2020; 369: 1318-1330. DOI: 10.1126/science.aaz1776.
2. Oh HS, Rutledge J, Nachun D, et al. Organ aging signatures in the plasma proteome track health and disease. *Nature* 2023; 624: 164-172. 20231206. DOI: 10.1038/s41586-023-06802-1.
3. Uhlén M, Fagerberg L, Hallström BM, et al. Proteomics. Tissue-based map of the human proteome. *Science* 2015; 347: 1260419. DOI: 10.1126/science.1260419.
4. Love MI, Huber W and Anders S. Moderated estimation of fold change and dispersion for RNA-seq data with DESeq2. *Genome Biol* 2014; 15: 550. DOI: 10.1186/s13059-014-0550-8.
